# AMPK activation induced in pemetrexed‐treated cells is associated with development of drug resistance independently of target enzyme expression

**DOI:** 10.1002/1878-0261.12496

**Published:** 2019-05-15

**Authors:** Yiyang Qin, Ikuo Sekine, Michiko Hanazono, Takao Morinaga, Mengmeng Fan, Yuichi Takiguchi, Yuji Tada, Masato Shingyoji, Naoto Yamaguchi, Masatoshi Tagawa

**Affiliations:** ^1^ Division of Pathology and Cell Therapy Chiba Cancer Center Research Institute Japan; ^2^ Laboratory of Molecular Cell Biology Graduate School of Pharmaceutical Sciences Chiba University Japan; ^3^ Department of Medical Oncology Faculty of Medicine University of Tsukuba Japan; ^4^ Department of Respirology Graduate School of Medicine Chiba University Japan; ^5^ Department of Medical Oncology Graduate School of Medicine Chiba University Japan; ^6^ Division of Respirology Chiba Cancer Center Japan; ^7^ Department of Molecular Biology and Oncology Graduate School of Medicine Chiba University Japan

**Keywords:** AMP‐activated protein kinase, drug resistance, mesothelioma, mTORC1, pemetrexed

## Abstract

Pemetrexed (PEM) inhibits DNA and RNA synthesis and is currently one of the first‐line agents for mesothelioma. PEM suppresses the activities of several enzymes involved in purine and pyrimidine synthesis, and elevated activity of these enzymes in tumors is often linked with resistance to PEM. The agent also stimulates AMP‐activated protein kinase (AMPK) and consequently influences the mammalian target of rapamycin complex 1 (mTORC1) pathways. Nevertheless, it remains unclear whether PEM resistance is linked to the AMPK or mTORC1 pathways. Here, we established two independent PEM‐resistant mesothelioma cell lines in which expression of the PEM‐target enzymes was not elevated, and found that levels of phosphorylated AMPK and p70S6K and, to a lesser extent, levels of phosphorylated AKT and p53, were increased in these cells as compared with the respective parent cells. PEM stimulation also augmented phosphorylation of AMPK, p70S6K, AKT and p53 in most cases. An AMPK activator increased phosphorylation and PEM resistance in parental cells, and the inhibitor decreased the resistance of PEM‐resistant cells. In contrast, inhibitors for p70S6K and AKT did not influence PEM resistance; furthermore, increased levels of endogenous p53 did not affect PEM sensitivity. These data collectively indicate that constitutive activation of AMPK is associated with PEM resistance, and that this is unconnected with elevated DNA and RNA synthesis.

**Abbreviations**
4E‐BP1: eukaryotic translation initiation factor 4E‐binding protein 1
ACC: acetyl‐CoA‐carboxylate
AICART: aminoimidazolecarboxamide ribonucleotide formyltransferase
AMPK: AMP‐activated protein kinase
CDDP: cisplatin
DHFR: dihydrofolate reductase
GARFT: glycinamide ribonucleotide formyltransferase
mTORC1: mammalian target of rapamycin complex 1
PEM: pemetrexed
p70S6K: p70 ribosomal protein S6 kinase
TS: thymidylate synthase
ZMP: aminoimidazolecarboxamide ribonucleotide

## 1 Introduction

Malignant mesothelioma, developed mainly in the pleural cavity, is often associated with occupational asbestos exposure (Robinson *et al*., 2005). Radical operations have failed to improve the prognosis, and radiotherapy is used primarily for a palliative purpose (Yap *et al*., 2017). Most of the patients are therefore subjected to chemotherapy, and combination of cisplatin (CDDP) and pemetrexed (PEM) is currently the first‐line regimen. Responses to the chemotherapeutic agents are, however, poor, and the median survival period is about 12 months (Vogelzang *et al*., 2003). The patient often becomes resistant to the agents, and no effective second‐line drug is yet available (Bronte *et al*., 2016). A mechanism of the drug resistance is therefore a clue to improve efficacy of the first‐line agents for mesothelioma patients. A number of studies clarified mechanisms of acquired resistance to CDDP (Galluzzi *et al*., 2013; Kartalou and Essigmann, 2001) but how PEM resistance was developed was not well understood.

Pemetrexed inhibits DNA and RNA synthesis by suppressing activities of three major target enzymes: thymidylate synthase (TS), glycinamide ribonucleotide formyltransferase (GARFT) and dihydrofolate reductase (DHFR). These are primary targets of PEM and are involved in the folate metabolic pathway, which plays a critical role in purine and pyrimidine synthesis (Shih *et al*., 1994). Moreover, expression levels of these enzymes in tumors were linked with PEM resistance (Flynn *et al*., 2006; Takezawa *et al*., 2011). We previously established PEM‐resistant mesothelioma cells with a stepwise increase of PEM concentrations and found that some of the PEM‐resistant cells increased expression of TS and GARFT in comparison with the respective parent cells, whereas others did not have such a differential expression of these enzymes (Kitazono‐Saitoh *et al*., 2012). The second target of PEM is aminoimidazolecarboxamide ribonucleotide formyltransferase (AICART), a folate‐dependent enzyme involved in *de novo* purine synthesis. PEM‐treated cells consequently accumulated an AICART substrate, aminoimidazolecarboxamide ribonucleotide (ZMP), and the substrate stimulated AMP‐activated protein kinase (AMPK), since ZMP was an analog of AMP (Racanelli *et al*., 2009; Rothbart *et al*., 2010). AMPK is a heterotrimeric complex consisting of three molecules, α‐, β‐ and γ‐subunits, and activation of AMPK is mediated by phosphorylated AMPKα (Jeon and Hay, 2015). Activated AMPK inhibited the mammalian target of rapamycin complex 1 (mTORC1) pathway which mediated cellular functions through eukaryotic translation initiation factor 4E‐binding protein 1 (4E‐BP1) and p70 ribosomal protein S6 kinase (p70S6K) (Racanelli *et al*., 2009). Moreover, ZMP activated the AKT pathway and AKT reciprocally interacted with AMPK (Kuznetsov *et al*. 2011; Rothbart *et al*., 2010). These data collectively indicated that PEM also influenced the AMPK, mTORC1 and AKT pathways, in addition to DNA and RNA synthesis. How PEM achieved anti‐tumor effects through the pathways remained uncharacterized, and any possible role of the AMPK, mTORC1 and AKT in PEM resistance was not investigated. On the other hand, AMPK phosphorylated p53 tumor suppressor, which was associated with cellular susceptibility to an anti‐cancer agent, and p53 reciprocally activated AMPK (Jones *et al*., 2005; Li *et al*., 2015). In addition, PEM induced DNA damage and activated the p53 pathways (Buqué *et al*., 2012). These data also suggested that p53 played a certain role in AMPK activation and PEM sensitivity.

In this study, we used the PEM‐resistant mesothelioma cells of which *TS* and *GARFT* transcript levels were rather lower than those of respective parent cells, clarified how PEM influenced AMPK. mTORC1, AKT and p53 expression, and investigated a possible contribution of these pathways to PEM resistance.

## 2 Materials and methods

### 2.1 Cells and agents

Human mesothelioma cells, NCI‐H28, NCI‐H226, MSTO‐211H and NCI‐H2452, and immortalized cells of mesothelium origin, Met‐5A, were purchased from American Type Culture Collection (Manassas, VA, USA). Mesothelioma with mutated *p53* genotype, EHMES‐1 and JMN‐1B cells were provided by Dr. Hironobu Hamada (Hiroshima University, Japan) (Nakataki *et al*., 2006). PEM‐resistant H28‐PEM, H226‐PEM, 211H‐PEM, and H2452‐PEM cells were previously established from the respective parent cells by a stepwise increase of PEM. CDDP‐resistant NCI‐H28, MSTO‐211H and NCI‐H2452 cells were also established with the same method (Kitazono‐Saitoh *et al*., 2012). Cells were cultured with RPMI‐1640 medium supplemented with 10% fetal calf serum and confirmed to be negative for mycoplasma. The genotype of *p53* was wild‐type in NCI‐H28, NCI‐H226, MSTO‐211H and NCI‐H2452 cells, but p53 protein of NCI‐H2452 cells was truncated (Di Marzo *et al*., 2014). Chemicals used in the present study were purchased as follows: PEM (Eli Lilly, Indianapolis, IN, USA), A769662 (Catalogue number: ab120335), PF4708671 (ab141993), compound C (ab120843, Abcam, Cambridge, UK), nutlin‐3a (S8059, Selleck, Houston, TX, USA), MK‐2206 (CT‐MK2206, ChemieTek, Indianapolis, IN, USA) and rapamycin (R8781, Sigma‐Aldrich, St. Louis, MO, USA).

### 2.2 Cell viability test

Cells seeded in 96‐well plates (2.8 × 10^3^ cells per well) were treated with an agent and incubated with PEM for 72 h. Cell viabilities were assessed with a WST‐8 kit (Dojindo, Kumamoto, Japan) and the relative viability was calculated based on the absorbance at 450 nm without any treatments (WST assay). Half maximal inhibitory concentration (IC_50_) values were estimated with calcusyn software (Biosoft, Cambridge, UK) based on the WST assay. The statistical analysis was performed with one‐way analysis of variance.

### 2.3 Western blot analysis

Cell lysate was subjected to sodium dodecyl sulfate‐polyacrylamide gel electrophoresis. The protein was transferred to a nitrocellulose membrane and hybridized with antibody against AMPKα (catalog number: #2532), phosphorylated AMPKα at Thr 172 (#2535), 4E‐BP1 (#9452), phosphorylated 4E‐BP1 at Thr 37/46 (#9459), p70S6K (#9202), phosphorylated p70S6K at Thr389 (#9205), phosphorylated p53 at Ser 15 (#9284), AKT (#9272), phosphorylated AKT at Ser 473 (#9271), ACC at Ser 79 (#3661), actin (#4970) (Cell Signaling, Danvers, MA, USA), phosphorylated H2AX at Ser 139 (#613401) (BioLegend, San Diego, CA, USA), p53 (Ab‐6, clone DO‐1) and tubulin‐α (clone DM1A, Thermo Fisher Scientific, Fremont, CA, USA) followed by an appropriate second antibody. The membranes were developed with the ECL system (GE Healthcare, Buckinghamshire, UK). Actin and tubulin‐α were used as a loading control. DMSO, a solvent of nutlin‐3a, was used as a control.

## 3 Results

### 3.1 Establishment of PEM‐resistant mesothelioma

**Figure 1:**
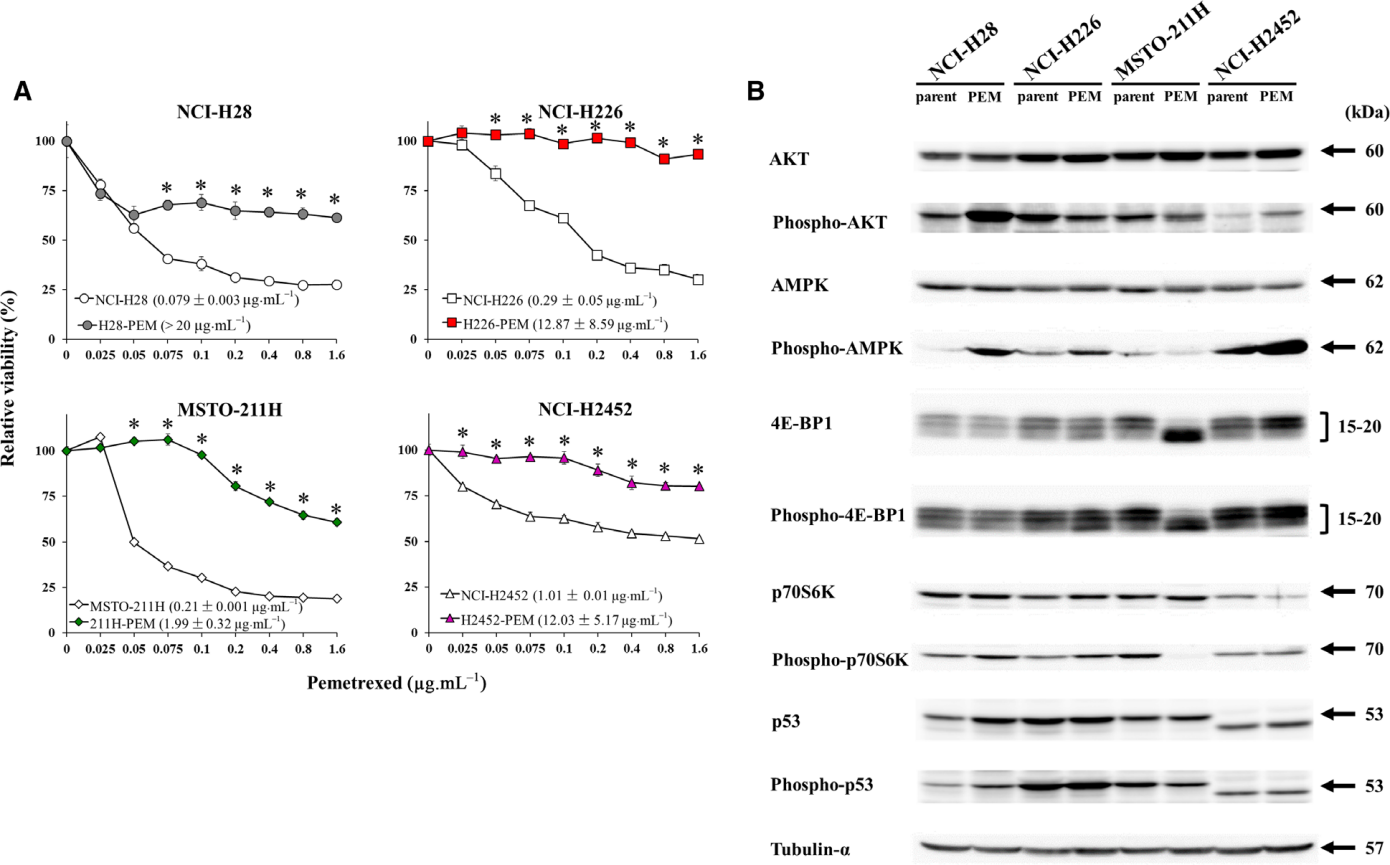
Cell viability and expression of AMPK and related molecules in parent and PEM‐resistant cells. (A) Paired cells, NCI‐H28/H28‐PEM, NCI‐H226/H226‐PEM, MSTO‐211H/211H‐PEM, and NCI‐H2452/H2452‐PEM cells, were treated with various concentrations of PEM as indicated for 72 h and cell viability was measured with the WST assay. Average and standard error bars are shown (*n* = 3). IC_50_ values with SE are shown as pg·mL^−1^ and asterisks indicate statistical significance of PEM sensitivity between parent and PEN‐resistant cells (*P* < 0.05, ANOVA). (B) Paired parent and PEM‐resistant cells were examined for expression levels of AMPK and the related molecules with Western blot analysis as indicated. Tubulin‐α was used as a loading control.

We established PEM‐resistant mesothelioma, H28‐PEM, H226‐PEM, 211H‐PEM and H2452‐PEM cells from the parent cells NCI‐H28, NCI‐H226, MSTO‐211H and NCI‐H2452, respectively, and showed the PEM resistance with a colony‐forming assay (Kitazono‐Saitoh *et al*., 2012). We then examined PEM sensitivity with a different method in the present study and confirmed the resistance. The colorimetric WST assay, testing the cell viability, demonstrated that these PEM‐resistant cells were less sensitive to PEM compared with the respective parent cells (Fig. 1A). IC_50_ values indicated decreased susceptibility of the resistant cells to PEM.

### 3.2 Molecular expression in paired cells

We previously reported that 211H‐PEM and H2452‐PEM cells elevated *TS* and *GARFT* transcripts in comparison with the respective parent cells, whereas H28‐PEM and H226‐PEM cells did not up‐regulate transcripts of the PEM‐related enzymes including *DHFR*, and the expression levels were rather lower than those of parent cells (Kitazono‐Saitoh *et al*., 2012). We therefore sought a possible mechanism of the PEM resistance that was not related to the DNA and RNA synthesis and examined expression of the second PEM‐target, AMPK and the related molecules, with H28‐PEM and H226‐PEM cells. In the present study we compared expression levels of AMPKα, AKT, 4E‐BP1, p70S6K and p53 between the paired cells (Fig. 1B, Table S1). AMPKα expression remained the same between the paired cells, but phosphorylated AMPKα level was greater in H28‐PEM and H226‐PEM cells than in their parent cells. The phosphorylation level in H2452‐PEM cells also increased in comparison with that in the parent cells, but the level in 211H‐PEM cells was lower than that in the parent cells. Activation of AMPK evidenced by the phosphorylation led inhibition of the mTORC1 pathway, which was associated with dephosphorylation of 4E‐BP1 and p70S6K. However, H28‐PEM and H226‐PEM cells did not induce the dephosphorylation despite AMPK activation signals. Multiple bands of 4E‐BP1 and phosphorylated 4E‐BP1 represent the isotypes (Gingras *et al*., 1999), and the expression levels were the same in parent and PEM‐resistant cells, although 211H‐PEM cells showed a differential pattern of the isotype expression from the parent cells. Expression of p70S6K was not markedly different between the paired cells but phosphorylated p70S6K showed differential expression levels between them. H28‐PEM and H226‐PEM cells expressed phosphorylated p70S6K levels greater than those in the respective parent cells, whereas the phosphorylation was undetectable in 211H‐PEM cells. H2452‐PEM cells did not increase the phosphorylated p70S6K level but the relative intensity was augmented due to the decreased p70S6K expression. These data showed that elevated phosphorylation of AMPKα and p70S6K in PEM‐resistant cells was commonly shared in NCI‐H28 and NCI‐H226 cells.

Expression of AKT which interacted with AMPK and was activated with PEM was not different between the paired cells, whereas phosphorylated AKT was enhanced in H28‐PEM and H2452‐PEM cells in comparison with their parent cells. The phosphorylation in H226‐PEM and 211H‐PEM cells was rather down‐regulated. We also examined p53 and the phosphorylation levels, since PEM induced DNA damage. Expression of p53 and the phosphorylation increased in H28‐PEM cells compared with the parent cells, but other cells did not show up‐regulated expression. NCI‐2452 and the PEM‐resistant cells expressed truncated p53 with a lower molecular weight than the authentic one, as previously reported (Di Marzo *et al*., 2014). These data collectively indicated that enhanced phosphorylation of AMPKα and p70S6K could be linked with a mechanism of PEM resistance, whereas phosphorylated AKT and p53 activation were unrelated to the resistance or were associated with a cell type‐specific mechanism of the resistance.

We also examined differential expression of AMPKα between CDDP‐resistant cells and the parent cells (Fig. S1, Table S1). These CDDP‐resistant cells were also established from the same parent cells and did not show cross‐resistance to PEM (Kitazono‐Saitoh *et al*., 2012). Expression of AMPKα and phosphorylated AMPKα was not different between the paired cells except CDDP‐resistant NCI‐H2452 cells, which decreased phosphorylated AMPKα in comparison with the parent cells. We also found that phosphorylated AKT was augmented in all the CDDP‐resistant cells; however, the reason for this currently remains unclear. These data indicated that increased AMPKα phosphorylation was not a general marker for drug resistance in mesothelioma but suggested that constitutive phosphorylation of AMPKα was linked with PEM resistance.

### 3.3 Effects of PEM treatment on AMPK, mTORC1 and AKT expression

**Figure 2:**
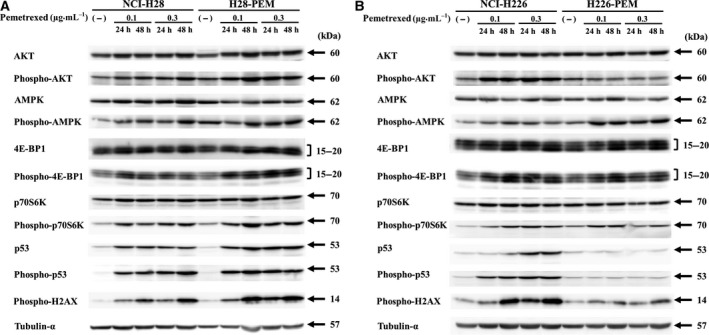
Expression of AMPK and related molecules in PEM‐resistant and parent cells. (A) NCI‐H28 and H28‐PEM cells or (B) NCI‐H226 and H226‐PEM cells were treated with PEM as indicated for 24 or 48 h, and the cell lysate was subjected to Western blot analysis. Tubulin‐α was used as a loading control.

We examined expression levels of AKT, AMPKα, 4E‐BP1, p70S6K and p53 in the paired cells derived from NCI‐H28 and NCI‐H226 cells (Fig. 2). Both NCI‐H28 and to a less extent H28‐PEM cells augmented phosphorylated AKT and AMPKα levels upon the PEM treatment, whereas there was only a minimal change in AKT and AMPKα levels (Fig. 2A, Table S1). AMPK activation in general induced dephosphorylation of 4E‐BP1 and p70S6K, but PEM‐treated mesothelioma cells rather showed increase of the phosphorylation; 4E‐BP1 and p70S6K levels were marginally influenced. We also found that PEM increased phosphorylated H2AX, a DNA damage marker, p53 and phosphorylated p53 levels in both cells. These data indicated that PEM induced activation of AKT, AMPK and p53 pathways as well as DNA damage but did not suppress the mTORC1 pathway.

We also examined expression of these molecules in NCI‐H226 and H226‐PEM cells (Fig. 2B, Table S1). NCI‐H226 cells showed the same responses to PEM as NCI‐H28 and H28‐PEM cells did. PEM‐treated NCI‐H226 cells showed augmentation of phosphorylated AKT, AMPKα, 4E‐BP1 and p70S6K levels but did not induce significant changes in the expression of the total proteins. In addition, phosphorylated H2AX, p53 and the phosphorylated p53 levels increased upon PEM treatment. In contrast, H226‐PEM cells did not up‐regulate expression of phosphorylated AKT but increased that of phosphorylated AMPKα, 4E‐BP1 and p70S6K. Expression levels of p53 and the phosphorylated p53 in H226‐PEM cells remained unchanged with PEM treatment despite a minor increase in phosphorylated H2AX levels. The poor responses of AKT, p53 and H2AX in H226‐PME cells were probably attributable to greater PEM resistance than in H28‐PEM cells (Fig. 1A). We then examined the expression levels in H226‐PEM cells with a high PEM concentration (Fig. S2). The cells that experienced more DNA damage increased phosphorylation of H2AX, p53 and AKT. H226‐PEM cells treated with a high PEM dose thus stimulated AKT, and p53 pathways as observed in PEM‐treated other cells. These data collectively indicated that PEM treatment induced activation of AKT, AMPK and p53 but did not inhibit mTORC1 pathways. Responses of these molecules in PEM‐treated cells were not direct evidence of a link with PEM resistance, but did imply an involvement of the pathways in the resistance.

### 3.4 AMPK activation promoted PEM resistance

**Figure 3:**
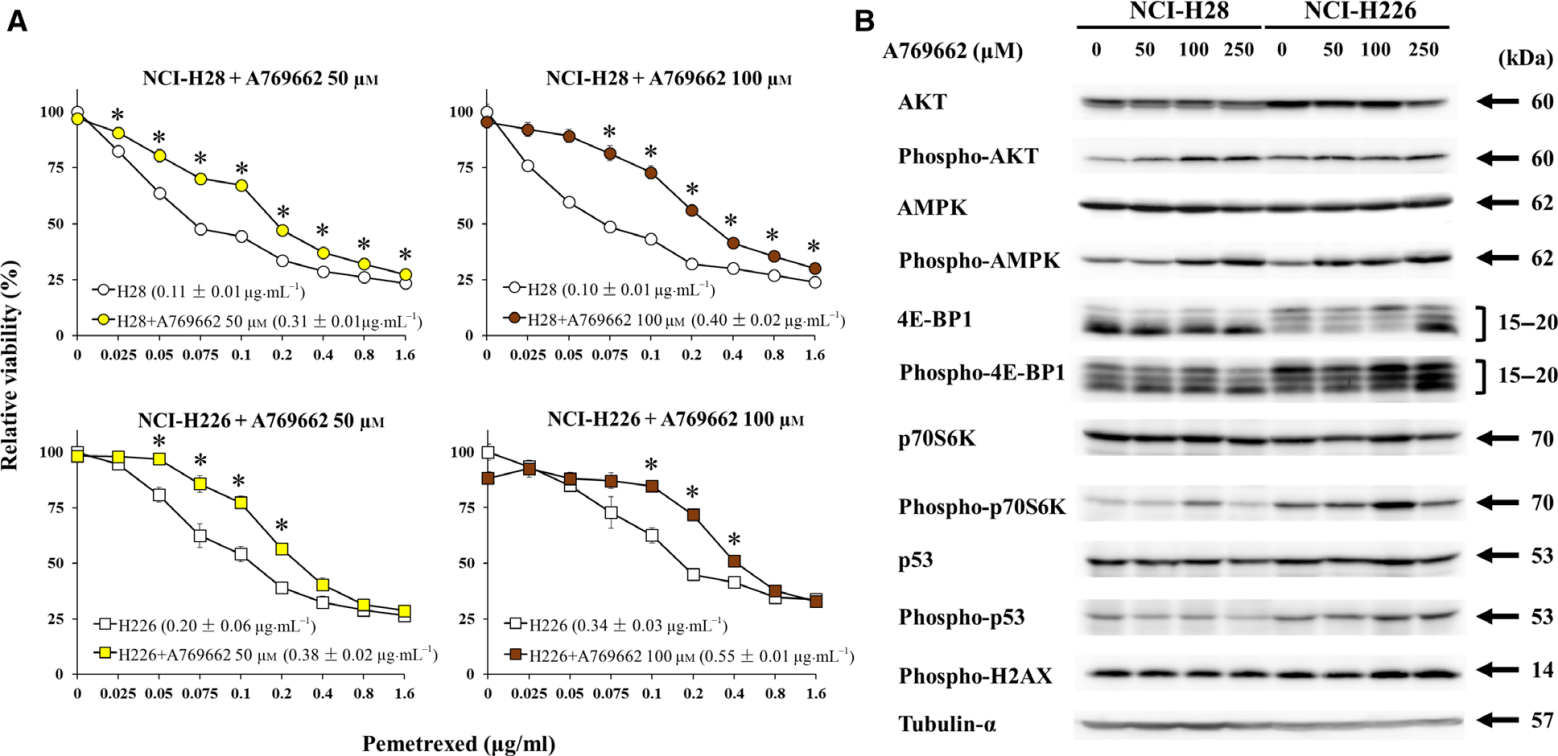
AMPK activation decreased PEM sensitivity. (A) NCI‐H28 and NCI‐H226 cells treated with or without A769662 were further incubated with PEM as indicated and the viability was assayed with the WST assay. Average and standard error bars are shown (*n* = 3). IC_50_ values with SE are shown as pg·mL^−1^, and asterisks indicate statistical significance of PEM sensitivity between parent and PEN‐resistant cells (*P* < 0.05, ANOVA). (B) Western blot analysis on expression levels of AMPK and related molecules. Cells were treated with A769662 as indicated for 48 h. Tubulin‐α was used as a loading control.

We first investigated a possible role of activated AMPK in the development of PEM resistance. We examined whether activated AMPK increased PEM resistance with A769662, an AMPK activating agent (Fig. 3A). NCI‐H28 and NCI‐H226 cells were treated with A769662 and examined for the susceptibility to PEM. A769662 at 50 and 100 μm produced little effect on the viability, but both parent cells treated with the AMPK activator increased resistance to PEM. IC_50_ values of A769662‐treated cells were greater than those of untreated cells. We next examined the effects of A769662 on AMPK and the related pathways (Fig. 3B, Table S1). NCI‐H28 and NCI‐H226 cells treated with A769662 for 72 h at 250 μm decreased the viability (data not shown), but not for 48 h. We then treated the cells up to 48 h and examined the expression levels with Western blot analysis (Fig. S3). A769662‐treated cells increased phosphorylation of AMPK in both cells but up‐regulated p70S6K phosphorylation was transient in cells treated at 100 μm. Phosphorylated AKT increased in NCI‐H28 but not in NCI‐H226 cells, although the AKT level in NCI‐H226 cells decreased. In addition, phosphorylation of p53 and H2AX was marginally up‐regulated only in NCI‐H226 cells. One of the 4E‐PB1 isotypes increased the expression in NCI‐H226 cells treated at 250 μm, but the phosphorylation of all the isotypes scarcely increased. AMPK stimulation thereby did not decrease p70S6K phosphorylation, but activation of AKT and p53 pathways, and phosphorylation of 4E‐BP1 were dependent on the cell types. These data suggested that development of PEM resistance was associated with increased AMPK phosphorylation without a decrease in p70S6K phosphorylation. In contrast, increase of phosphorylated AKT, p53 and 4E‐BP1 caused by AMPK activation was cell type‐specific and might be involved in PEM resistance of individual cells.

**Figure 4:**
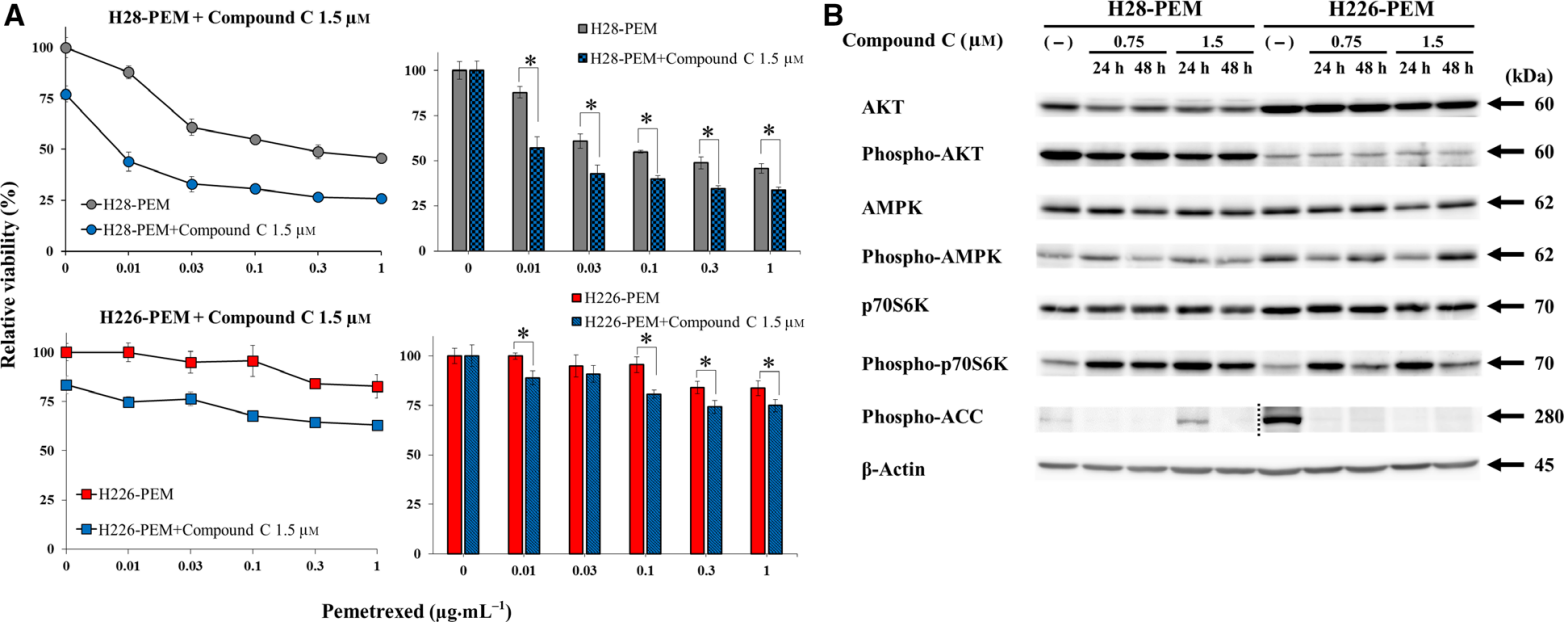
AMPK inactivation decreased PEM resistance. (A) H28‐PEM and H226‐PEM treated with or without compound C (1.5 μm) were further incubated with PEM as indicated and the viability was assayed with the WST assay. The bar graphs on the right showed relative viability that was calculated based on the viability of cells untreated or treated with compound C at 1.5 μm alone. Standard error bars are also shown (*n* = 3). *P < 0.05 (ANOVA). (B) Western blot analysis on expression levels of AMPK and related molecules in cells which were treated with compound C as indicated. β‐Actin was used as a loading control. A dotted line shows that blots of H28‐PEM and H226‐PEM cells were separately conducted due to differential expression levels of phosphorylated ACC between the cells.

We next examined whether an AMPK inhibitor, compound C, suppressed the PEM resistance of H28‐PEM and H226‐PEM (Fig. 4A). H28‐PEM and H226‐PEM cells were treated or untreated with compound C and then incubated with PEM. Inhibitory effects of compound C on PEM resistance were shown as the percent viability of cells relative to that of PEM‐untreated cells, since compound C by itself was cytotoxic to cells. Cells treated with compound C further decreased viability compared with those with PEM alone, indicating that the AMPK inhibitor increased PEM sensitivity. We then examined the effects of compound C on AKT, AMPK and p70S6K expression (Fig. 4B, Table S1). AKT phosphorylation remained unchanged but AMPK phosphorylation was inconsistent, depending on the time and the concentration used. Compound C did not directly inhibit an AMPK phosphorylating kinase activity, and dephosphorylation of AMPK was consequently not an indicator for AMPK inhibition in the cells. We then examined phosphorylation of acetyl‐CoA‐carboxylate (ACC), a substrate of AMPK and a marker for AMPK suppression (Zhang *et al*., 2017); compound C suppressed expression of phosphorylated ACC in H226‐PEM and, to less extent, H28‐PEM cells. The ACC phosphorylation in H28‐PEM cells was transiently up‐regulated but at present we do not know the reason for this. Compound C induced p70S6K phosphorylation in H28‐PEM and H226‐PEM cells. AMPK inhibition in general activated mTORC1 pathways and compound C consequently mediated increase of p70S6K phosphorylation. A769662 and compound C influenced PEM sensitivity in opposite ways, but p70S6K phosphorylation was up‐regulated in both cases, suggesting that phosphorylation of p70S6K was unrelated to PEM sensitivity.

### 3.5 Activated p70S6K was unrelated to PEM resistance

**Figure 5:**
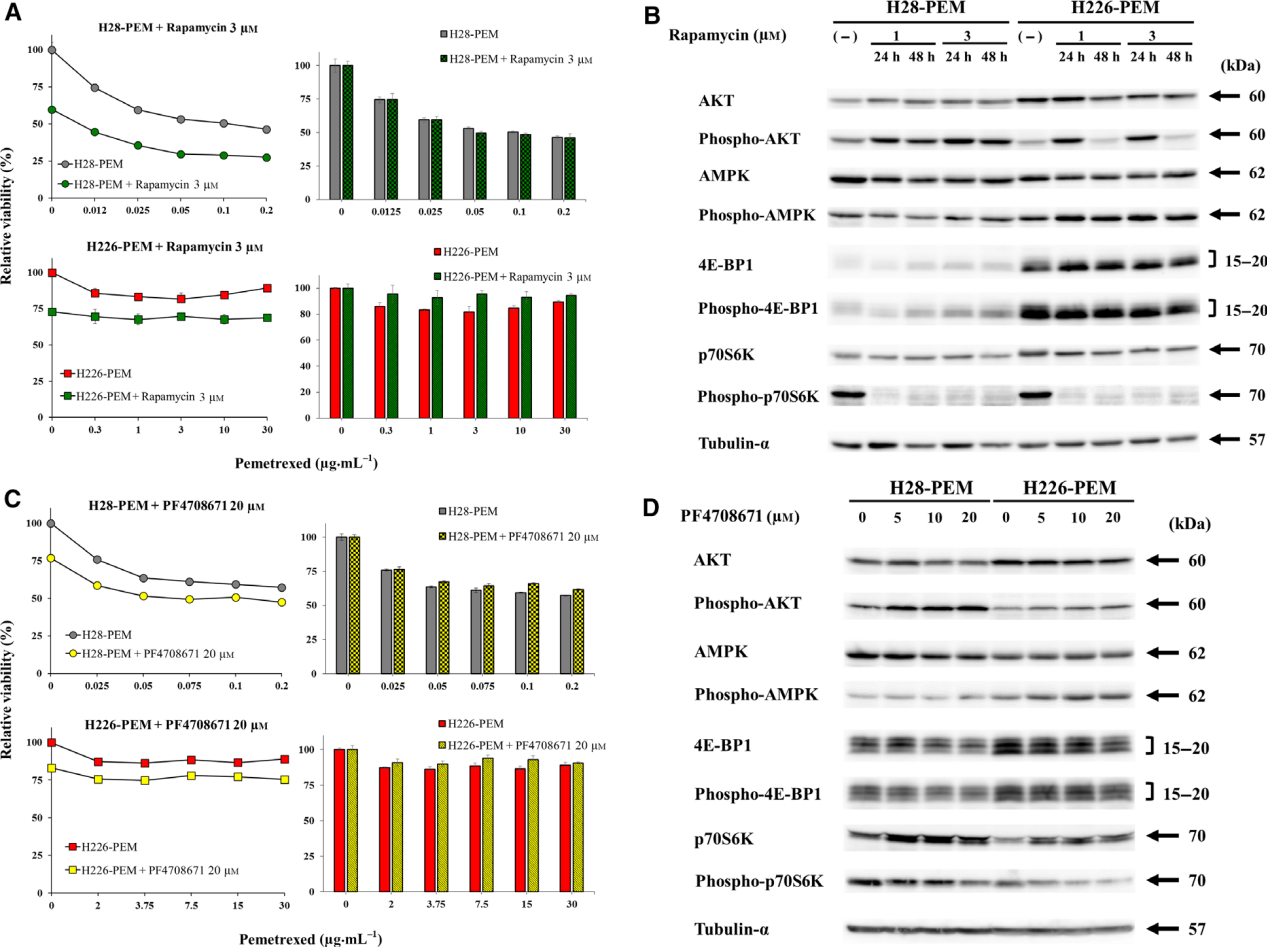
Inhibition of p70S6K was not associated with PEM resistance. (A) Cells treated with or without rapamycin (3 μm) were further incubated with PEM as indicated and the viability was assayed with the WST assay. The bar graphs on the right showed relative viability that were calculated based on the absorbance of cells untreated or treated with rapamycin at 3 μm alone. Standard error bars are also shown (*n* = 3). (B) Western blot analysis of cells treated with rapamycin as indicated. Tubulin‐α was used as a loading control. (C) Cells treated with or without PF4708671 (20 μm) were further incubated with PEM as indicated and the viability was assayed with the WST assay. The bar graphs on the right showed relative viability that were calculated based on the absorbance of cells untreated or treated with PF4708671 at 20 μm alone. Standard error bars are also shown (*n* = 3). Standard error bars are also shown (*n* = 3). (D) Western blot analysis of cells treated with PF4708671 as indicated. Tubulin‐α was used as a loading control.

We further examined a possible involvement of p70S6K in PEM resistance with the inhibitors. AMPK activation was linked with suppressed mTORC1 pathway, but the current study showed that expression of phosphorylated p70S6K was rather augmented in PEM‐resistant cells and did not decrease in A769662‐ or compound C‐treated cells. We thereby treated PEM‐resistant cells with the inhibitors rapamycin or PF4708671, and examined the PEM sensitivity (Fig. 5A). Rapamycin by itself inhibited viability of H28‐PEM and H226‐PEM, but did not affect PEM sensitivity of H28‐PEM cells or increased PEM resistance in H226‐PEM cells. Both H28‐PEM and H226‐PEM cells treated with rapamycin showed decreased phosphorylated p70S6K levels but minimally influenced expression of p70S6K (Fig. 5B, Table S1). Rapamycin‐mediated effects on AMPK and 4E‐BP1 were marginal, with a slight increase of AMPK phosphorylation in H226‐PEM. In contrast, AKT phosphorylation increased in H28‐PEM cells and temporally in H226‐PEM cells. These data indicated that rapamycin suppressed p70S6K activity and activated AKT, probably through a feedback mechanism in a cell type‐dependent manner, but had few effects on the AMPK and the 4E‐BP1 pathways. We also examined PF4708671, a different type of p70S6K inhibitor, and showed that the agent decreased viability of H28‐PEM and H226‐PEM cells but did not influence the PEM sensitivity (Fig. 5C). PF4708671 decreased p70S6K phosphorylation levels despite increased p70S6K expression, probably due to compensation for decreased p70S6K activity (Fig. 5D, Table S1). The inhibitor did not influence 4E‐BP1 levels but increased phosphorylation of AKT and AMPK, in particular in H28‐PEM cells. These data showed that PF4708671 was specific to p70S6K and reciprocally augmented AMPK and AKT activities due to a feedback effect, as we detected the same changes in rapamycin‐treated cells. These data collectively demonstrated that p70S6K activation was unrelated to the PEM resistance and that up‐regulated AKT or AMPK phosphorylation under suppressed p70S6K condition did not contribute to the PEM resistance.

### 3.6 AKT activation was unrelated to PEM resistance

**Figure 6:**
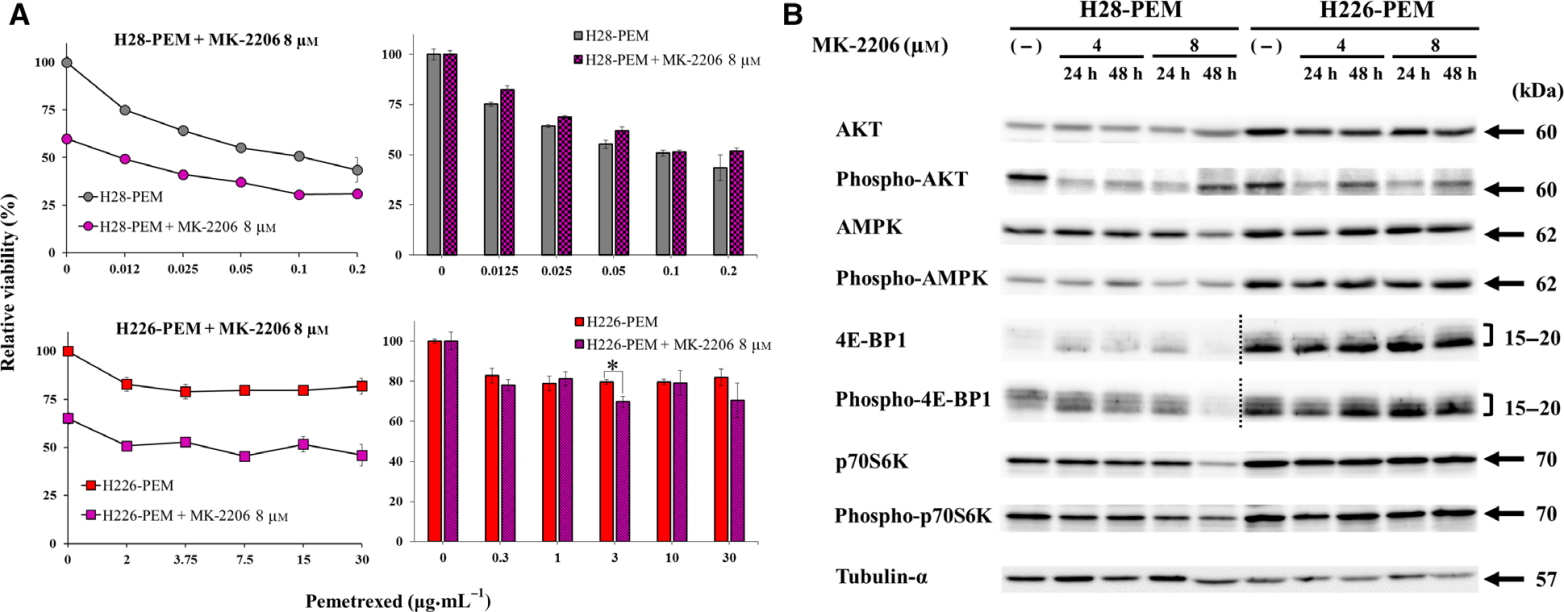
Inhibition of AKT was not associated with PEM resistance. (A) Cells treated with or without MK‐2206 (8 μm) were further incubated with PEM as indicated and the viability was assayed with the WST assay. The bar graphs on the right showed relative viability that were calculated based on the absorbance of cells untreated or treated with MK‐2206 at 8 μm alone. Standard error bars are also shown (*n* = 3). *P < 0.05 (ANOVA). (B) Western blot analysis of cells treated with MK‐2206 as indicated. Tubulin‐α was used as a loading control. Dotted lines show that exposure time of blots derived from H28‐PEM cells (long exposure) and H226‐PEM cells (short exposure) was different because of the differential expression levels of 4E‐BP1 and phosphorylated 4E‐BP1 between the cells.

We also examined a possible involvement of AKT activation in PEM resistance. AKT activation in general suppressed AMPK, but the current study showed that H28‐PEM cells showed up‐regulated AKT phosphorylation with augmented phosphorylation of AMPK (Fig. 1B) and that A769662, an AMPK activator, phosphorylated AKT in H28‐PEM cells (Fig. 3B). We therefore investigated effects of an AKT inhibitor, MK‐2206, on PEM susceptibility with PEM‐resistant cells (Fig. 6A). The inhibitor was cytotoxic to PEM‐resistant cells but did not influence PEM sensitivity in H28‐PEM cells or H226‐PEM cells except at 3 μm (Fig. 6A). Both cells treated with MK‐2206 decreased phosphorylated AKT with variable levels but did not influence AMPK phosphorylation (Fig. 6B, Table S1). The treatment did not affect phosphorylation of 4E‐BP1 or p70S6K in H226‐PEM cells but did decrease the phosphorylation in H28‐PEM cells. These data collectively showed that AKT inhibition did not influence PEM sensitivity and indicated that AKT activation was unrelated to PEM resistance.

### 3.7 Relationship between p53 activation and PEM resistance

**Figure 7:**
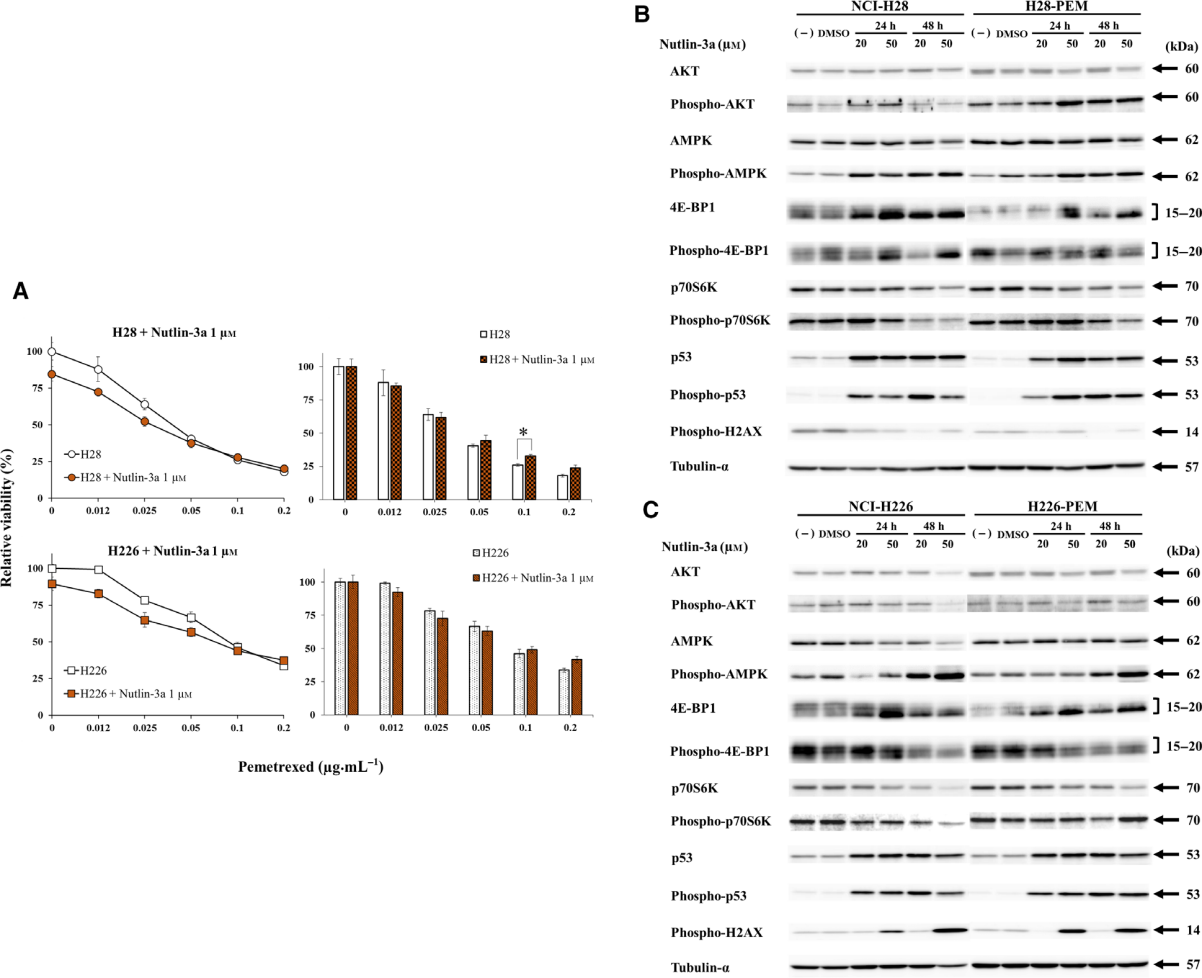
Augmented p53 expression was not associated with PEM resistance. (A) Cells treated with or without nutlin‐3a (1 μm) were further incubated with PEM as indicated and the viability was assayed with the WST assay. The bar graphs on the right showed relative viability that were calculated based on the absorbance of cells untreated or treated with nutlin‐3a at 1 μm alone. Standard error bars are also shown (*n* = 3). *P < 0.05 (ANOVA). (B) Western blot analysis of NCI‐H28 and H28‐PEM (B) or NCI‐H226 and H226‐PEM (C) cells treated with DMSO as a control as indicated. Tubulin‐α was used as a loading control.

We next investigated whether stimulation of the p53 pathway influenced AKT, AMPK and mTORC1 pathways and consequently affected PEM sensitivity. NCI‐H28 and NCI‐H226 cells with the wild‐type *p53* genotype and the PEM‐resistant cells were treated with nutlin‐3a to augment endogenous p53 expression (Fig. 7). Nutlin‐3a inhibited a binding between wild‐type p53 and MDM2 molecules with a p53 ubiquitination activity and subsequently enhanced p53 expression through decreased p53 degradation but not DNA damage. We tested PEM sensitivity in cells treated with nutlin‐3a (Fig. 7A). Nutlin‐3a suppressed viability of NCI‐H28 and NCI‐H226 cells but did not affect the PEM resistance except in NCI‐H28 cells treated with 0.1 μg·mL^−1^. We then examined molecular changes caused by nutlin‐3a‐mediated increase of p53 levels (Fig. 7B,C). The nutlin‐3a‐induced p53 phosphorylation was not associated with DNA damage because phosphorylated H2AX was not induced in NCI‐H28 and H28‐PEM cells (Fig. 7B). The up‐regulation of p53 augmented AKT phosphorylation in H28‐PEM, enhanced AMPK phosphorylation in NCI‐H28 and H28‐PEM cells, and decreased expression of p70S6K and the phosphorylation in NCI‐H28 cells ( Table S1). Effects of nutlin‐3a on 4E‐BP1 were minimal compared with those of control DMSO and induced differential expression levels depending on the isotypes. AMPK phosphorylation and p70S6K dephosphorylation were thereby commonly induced in NCI‐H28‐derived cells in which the p53 pathway was activated without DNA damage. On the other hand, nutlin‐3a to some extent induced different responses in NCI‐H226 and H226‐PEM cells (Fig. 7C). Expression levels of p53 and the phosphorylation increased in both cells and phosphorylation of H2AX were also induced at a high concentration at 50 μm. AKT phosphorylation remained unchanged but AMPK phosphorylation was augmented with the exception of NCI‐H226 cells treated with 20 μm for 24 h (Table S1). Nutlin‐3a decreased phosphorylated p70S6K levels in NCI‐H226 cells but a relative ratio of phosphorylated p70S6K to the total protein increased, and nutlin‐3a treatment of H226‐PEM cells produced similar changes. Phosphorylation of 4E‐BP1 decreased in nutlin‐3a‐treated NCI‐H226 and H226‐PEM cells, and increased 4E‐BP1 levels further down‐regulated the relation ratio of phosphorylated 4E‐BP1. These data showed that augmentation of p53 with nutlin‐3a increased AMPK phosphorylation and produced mixed responses in 4E‐BP1 and p70S6K phosphorylation, but did not influence AKT phosphorylation except in H28‐PEM cells. These data consequently indicated that p53 up‐regulation was not linked with PEM resistance but nutlin‐3a‐treated cells showed increase of AMPK phosphorylation similar to A769662. The nutlin‐3a treatment, however, produced different effects on p70S6K phosphorylation than did A769662 treatment.

We examined whether PEM‐induced phosphorylation of AMPK was associated with the *p53* genotype, since AMPK activation induced p53 phosphorylation and the enhanced p53 expression stimulated AMPK (Feng *et al*., 2005; Jones *et al*., 2005). Mesothelioma with mutated *p53* genotype, EHMES‐1 and JMN‐1B cells, and mesothelium‐derived Met‐5A cells expressing dominant‐negative p53, showed augmented phosphorylation of AMPK when they were treated with PEM (Fig. S4, Table S1). The treatment induced phosphorylation of H2XA in all the cells at 48 h, but AMPK phosphorylation in JMN‐1B and Met‐5A cells was enhanced even without DNA damage. These data indicated that activation of AMPK was not linked to the *p53* genotype or DNA damage. Phosphorylation of AKT was not enhanced in cells with mutated *p53* genotype or in those with loss of p53 functions, suggesting that the p53 pathways were involved in the PEM‐mediated AKT activation.

## 4 Discussion

The present study showed that PEM‐resistant mesothelioma cells which did not augment expression of PEM‐target enzymes for DNA and RNA synthesis constitutively up‐regulated phosphorylation of AMPK and p70S6K. We also demonstrated that an AMPK activator decreased PEM sensitivity and the inhibitor increased the sensitivity, whereas p70S6K inhibitors did not influence the PEM sensitivity. PEM resistance which developed in an independent manner of the primary targets can be therefore linked with AMPK activation, which is induced by inhibition of the second target of PEM – AICART.

A role of AMPK in cell proliferation is complex and can be pro‐survival or apoptotic depending on cell types and on the energy supply level in the cells (Li *et al*., 2015; Wang *et al*., 2016). AMPK activation, represented by the phosphorylation, can produce survival effects in resting cells but can induce cell death in those with increased anabolic rates (Kuznetsov *et al*., 2011). The activation in cancer cells in general supports cell proliferation, despite changing the energy metabolism (Jeon and Hay, 2015; Park *et al*., 2009). The AMPK functions in cancer were also regulated by multiple factors and were consequently subjected to cellular contexts. A possible involvement of AMPK in development of resistance to anti‐cancer agents can be linked to cellular energy levels (Budanov and Karin, 2008), but a direct contribution of AMPK stimulation to the drug resistance had not yet been shown. The present study demonstrated that PEM resistance was associated with constitutive AMPK activation. We established PEM‐resistant mesothelioma cells which did not augment expression of primary target enzymes of PEM and compared differential expression levels of the PEM secondary target and the related molecules. The PEM‐resistant cells derived from NCI‐H28 and NCI‐H226 cells constitutively increased phosphorylation of AMPK and p70S6K with varying levels of AKT, 4E‐BP1 and p53 phosphorylation. We demonstrated that an AMPK activator and the inhibitor increase and decrease the PEM sensitivity, respectively, whereas other agents influencing p70S6K, AKT and p53 phosphorylation did not. These non‐AMPK acting agents, however, also affected phosphorylation of AMPK and the related molecules because of a possible cross‐talk among these molecules. In fact, rapamycin and PF4708671 augmented AKT and AMPK phosphorylation depending on cells, and nutlin‐3a also increased phosphorylation levels of AMPK in all the cells and AKT in H28‐PEM cells. Moreover, A769662 phosphorylated AKT and to less of extent p70S6K, and increased p53 expression in NCI‐H226 cells. These data suggested that PEM resistance was not solely attributable to constitutive activation of AMPK. We therefore raised the possibility that phosphorylation of p70S6K or 4E‐BP1 might play a role in the AMPK‐mediated PEM resistance. The present study showed that cells treated with an AMPK activator or inhibitor did not show down‐regulated p70S6K or 4E‐BP1 phosphorylation, in contrast to those treated with rapamycin, PF4708971 or nutlin‐3a, all of which increased AMPK phosphorylation but down‐regulated either p70S6K or 4E‐BP1 phosphorylation. These results thereby suggested that development of PEM resistance was ascribable to constitutive activation of AMPK under a certain condition in which mTORC1 activity was uninhibited. Activation of AMPK often suppresses mTORC1 actions and in general leads to retarded cell growth and suppressed metabolism. We therefore assume that an uninhibited mTORC1 pathway in PEM‐resistant cells did not suppress growth‐related signals and maintained cell survival even under an activated AMPK condition. The present study suggested that activated AMPK in cancer was not linked to growth inhibition and that uninhibited mTORC1 rather supported proliferation of tumors, which resulted in the drug resistance. The assumption, however, did not rule out a possible involvement of non‐AMPK molecules in PEM resistance. It remained unknown how AMKP activation was compatible with enhanced phosphorylation of p70S6K and 4E‐BP1, and why the AMPK stimulator and the inhibitor similarly increased the p70S6K phosphorylation levels in the same cells. Complexity of the mTORC1 responses induced by AMPK may be attributable to an involvement of multiple factors which regulate the AMPK‐mTORC1 axis according to how AMPK activity is influenced. In addition, the mTORC1 itself was regulated by not only AMPK but by other pathways (Budanov and Karin, 2008), which could result in cell type‐ and context‐dependent mTORC1 responses.

AMPK reciprocally influences AKT actions through a cross‐talk between them (Kuznetsov *et al*., 2011; Rothbart *et al*., 2010). AMPK competes with AKT in mTORC1 regulations, and AKT activation suppresses AMPK functions. These interactive pathways indicated that stimulation with a single agent influenced multiple effects depending on the cellular contexts. PEM‐treated cells showed augmentation of AKT phosphorylation, which could be associated with enhanced 4E‐BP1 and p70S6K phosphorylation despite AMPK phosphorylation. AKT activation can block AMPK‐mediated dephosphorylation of p70S6K through direct activation of p70S6K, and a previous study also showed that activated AKT and mTORCl were overexpressed in PEM‐resistant osteosarcoma cells (Zhu *et al*., 2014). The current study, however, showed that an AKT inhibitor, MK‐2206, did not influence AMPK or p70S6K phosphorylation or PEM sensitivity. We also examined a role of p53 in AMPK activation in terms of a possible cross‐talk between them. Nutlin‐3a‐treated NCI‐H28, NCI‐H226 and H28‐PEM cells, bearing the wild‐type *p53* genotype, increased p53 without phosphorylated H2AX and all the treated mesothelioma cells increased AMPK phosphorylation. The AMPK activation was also induced with PEM even in cells bearing mutated *p53* genotype or expressing dominant p53 under no DNA damage. Previous studies showed that an increased p53 level activated AMPK and, furthermore, AMPK augmented the p53 levels, which indicated reciprocal interactions between AMPK and p53 pathways (Feng *et al*., 2005; Jones *et al*., 2005; Li *et al*., 2015). The present study, however, demonstrated that PEM increased AMPK phosphorylation irrespective of the *p53* genotypes and suggested that activated p53 pathways played a minor role in AMPK activation in mesothelioma. Moreover, the influence of *p53* genotype on PEM sensitivity remained unclear, since PEM‐mediated cell death was not always linked with the p53 pathway and PEM‐related enzyme activities were not controlled by the p53 pathways (Giovannetti *et al*., 2007; Yang *et al*., 2013).

A mechanism of elevated AMPK for the PEM resistance remained uncharacterized. AMPK is involved in a number of cellular events which can contribute to development of drug resistance (Wang *et al*., 2016). AMPK is activated by low ATP levels and converts metabolic pathways to support cell survival under a number of cellular stresses. PEM‐treated cells received DNA damage due to inhibited DNA and RNA synthesis and activated AMPK to maintain the cellular energy levels. Constitutive AMPK activation in PEM‐treated cells therefore can shift their metabolic pathways in favor of counteracting cell death. Moreover, AMPK can also play a role in reprogramming of cancer stem cells (Oliveras‐Ferraros *et al*., 2014) and in inducing autophagy, which strengthens cell survival by maintaining nutrient levels (Possik *et al*., 2014). The biological significance of AMPK activation in development of PEM resistance can thus be diversified among cells tested, and a mechanism for constitutive activation of AMPK and how the activation leads to PEM resistance are the next issues to be investigated. Nevertheless, the present study suggested a possible use of an AMPK inhibitor in a patient who became PEM‐resistant. Recent studies indicated that sunitinib, a multiple kinase inhibitor, directly bound to AMPK and inhibited the AMPK activity more than compound C (Borgdorff *et al*., 2014; Jeon and Hay, 2015; Laderoute *et al*., 2010). The agent is clinically in use for renal cell carcinoma (Jeon and Hay, 2015) and may improve the PEM sensitivity of patients who developed resistance due to upregulated AMPK. A convenient clinical diagnostic tool is thereby required to select these patients and to exclude those whose resistance to PEM is caused by elevated enzymes related to DNA and RNA synthesis.

## 5 Conclusions

Pemetrexed primarily inhibited enzymes responsible for DNA and RNA synthesis pathways and activated AMPK activity. Two independent PEM‐resistant mesothelioma cells which did not elevate expression of the primary target enzymes showed increased AMPK and p70S6K phosphorylation. An AMPK‐activating agent increased PEM resistance in the parent cells and an AMPK inhibitor improved PEM sensitivity of the PEM‐resistant cells. Inhibitors for p70S6K or AKT, and p53 upregulation, however, did not influence the PEM sensitivity. These non‐AMPK‐acting agents, with the exception of an AKT inhibitor, also augmented AMPK phosphorylation but down‐regulated phosphorylation of p70S6K or 4E‐BP1. These data therefore indicated that PEM resistance was linked to elevated AMPK activity with uninhibited mTORC1 pathway. The present study demonstrated that PEM resistance could be attributable to constitutive activation of AMPK, the secondary target of PEM.

## Conflict of interest

The authors declare that there is no conflict of interests in this research. We obtained a grant from Nichias Corporation. Nichias Corporation is not a pharmaceutical company but a company making industrial products for building, automobiles and pipes (see http://www.nichias.co.jp/). The grant is part of their mécénat activities, corporate sponsorship, with the aim to assist medical research about intractable cancer treatments. We are thereby connected to any employment, consultancy, patents, products in development or marketed products of the company.

## Author contributions

YQ, IS, MH and TM designed experimental protocols, prepared materials and conducted experiments. MF and YT established cells and analyzed expression of a number of gene expression. YQ, YT and MS analyzed the data and prepared the figures. NY and MT organized the experiments and examined the results. MT and YQ prepared the manuscript. All authors read and approved the final manuscript.

## Supporting information


**Fig. S1.** Expression of AKT and AMPK in CDDP‐resistant cells. Parent and CDDP‐resistant cells were examined for the expression with Western blot analysis as indicated. Tubulin‐α was used as a loading control.

**Fig. S2.** Molecular changes in H226‐PEM cells treated with a high concentration of PEM. H226‐PEM cells were treated with PEM as indicated for 24 or 48 h, and the cell lysate was subjected to Western blot analysis. Tubulin‐α was used as a loading control.

**Fig. S3.** Relative expression levels of AKT, AMPK, p70S6K, and the respective phosphorylated proteins. Expression of these molecules in Figure 3B was quantitated as shown in Table S1 and expressed in bar graphs. A relative ratio between phosphorylated and total protein was also shown.

**Fig. S4.** AMT and AMPK activation in PEM‐treated cells with mutated *p53* genotype. Mesothelioma cells were treated with PEM as indicated and the cell lysate was subjected to Western blot analysis. Tubulin‐α was used as a loading control.

**Table S1.** A relative expression level of major proteins in Western blots. Signal intensity of chemiluminescence was measured after subtraction of a background level with imagej software (National Institute of Health, Bethesda, MD, USA, available at https://imagej.nihgov/ij/index.html). Intensity is shown as an arbitrary unit standardized by control intensity (β‐actin or tubulin‐α).
