# Supplementary figures and images for: AMPK activation induced in pemetrexed‐treated cells is associated with development of drug resistance independently of target enzyme expression
Source: Mol Oncol. 2019 May 15;13(6):1419–32. doi: 10.1002/1878-0261.12496 (PMC6547620; doi:10.1002/1878-0261.12496)

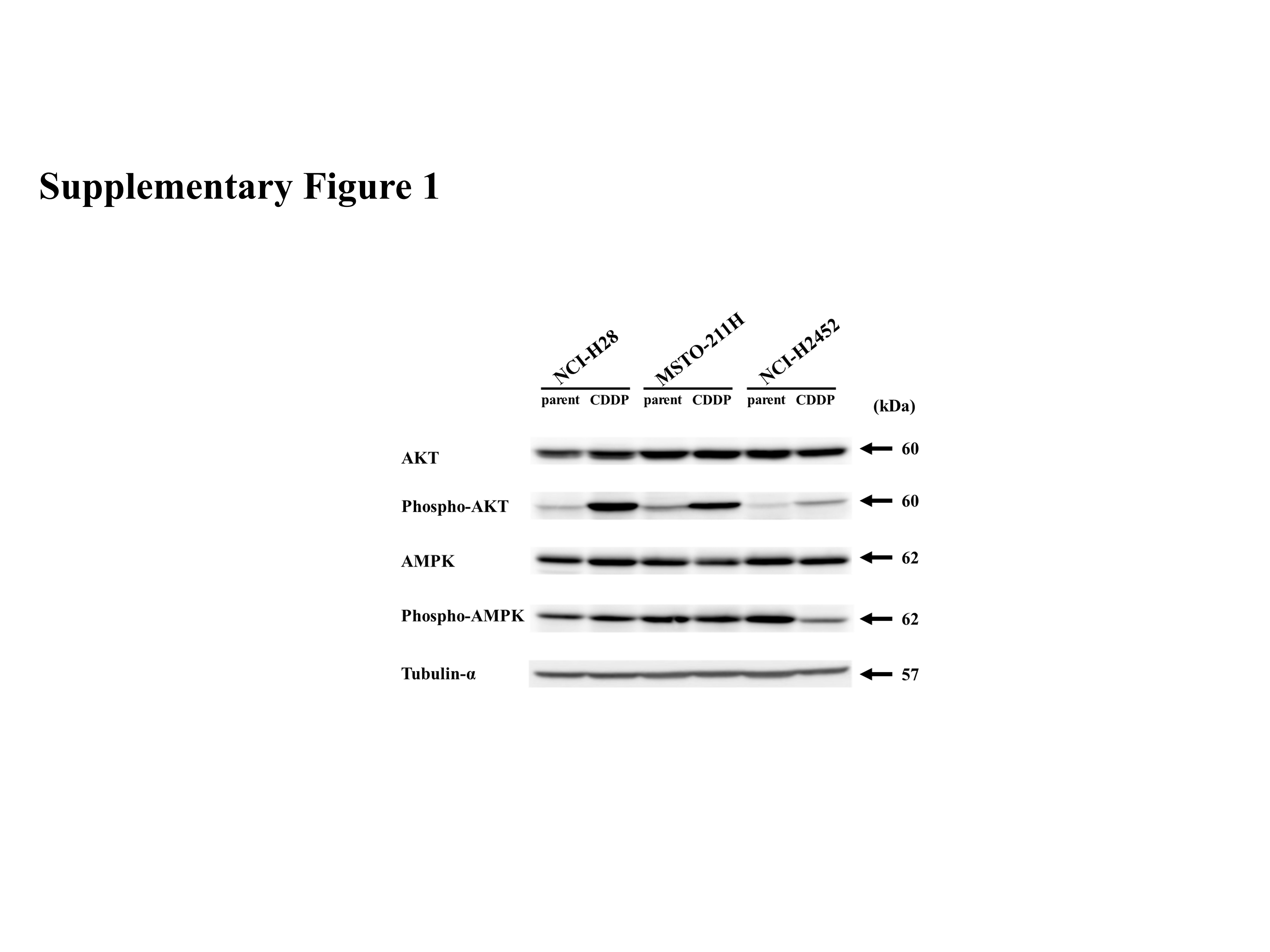

Supplement: Supplementary file 1 — Fig. S1. Expression of AKT and AMPK in CDDP‐resistant cells. Parent and CDDP‐resistant cells were examined for the expression with Western blot analysis as indicated. Tubulin‐α was used as a loading control. [file MOL2-13-1419-s001.TIF]

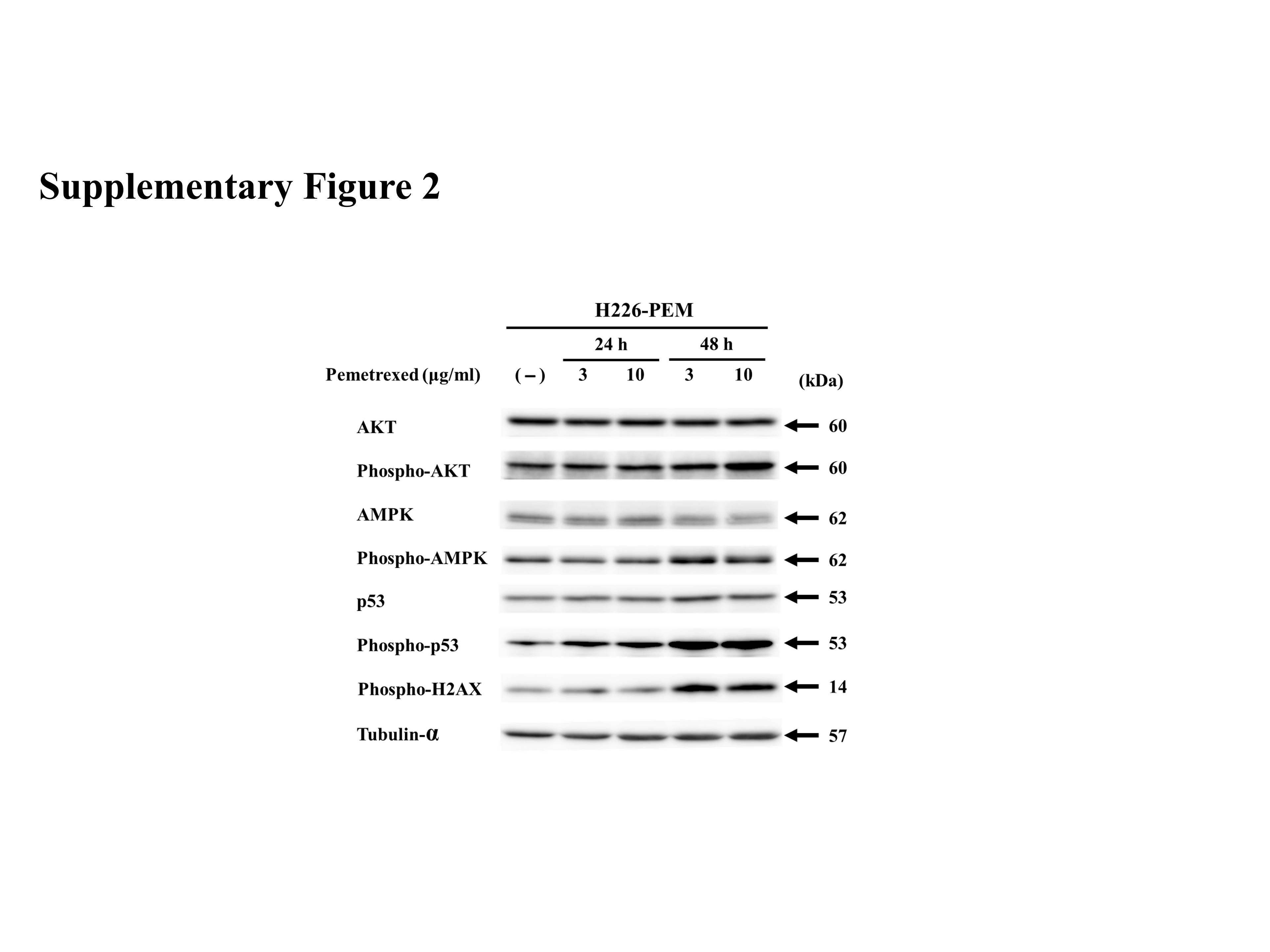

Supplement: Supplementary file 2 — Fig. S2. Molecular changes in H226‐PEM cells treated with a high concentration of PEM. H226‐PEM cells were treated with PEM as indicated for 24 or 48 h, and the cell lysate was subjected to Western blot analysis. Tubulin‐α was used as a loading control. [file MOL2-13-1419-s002.TIF]

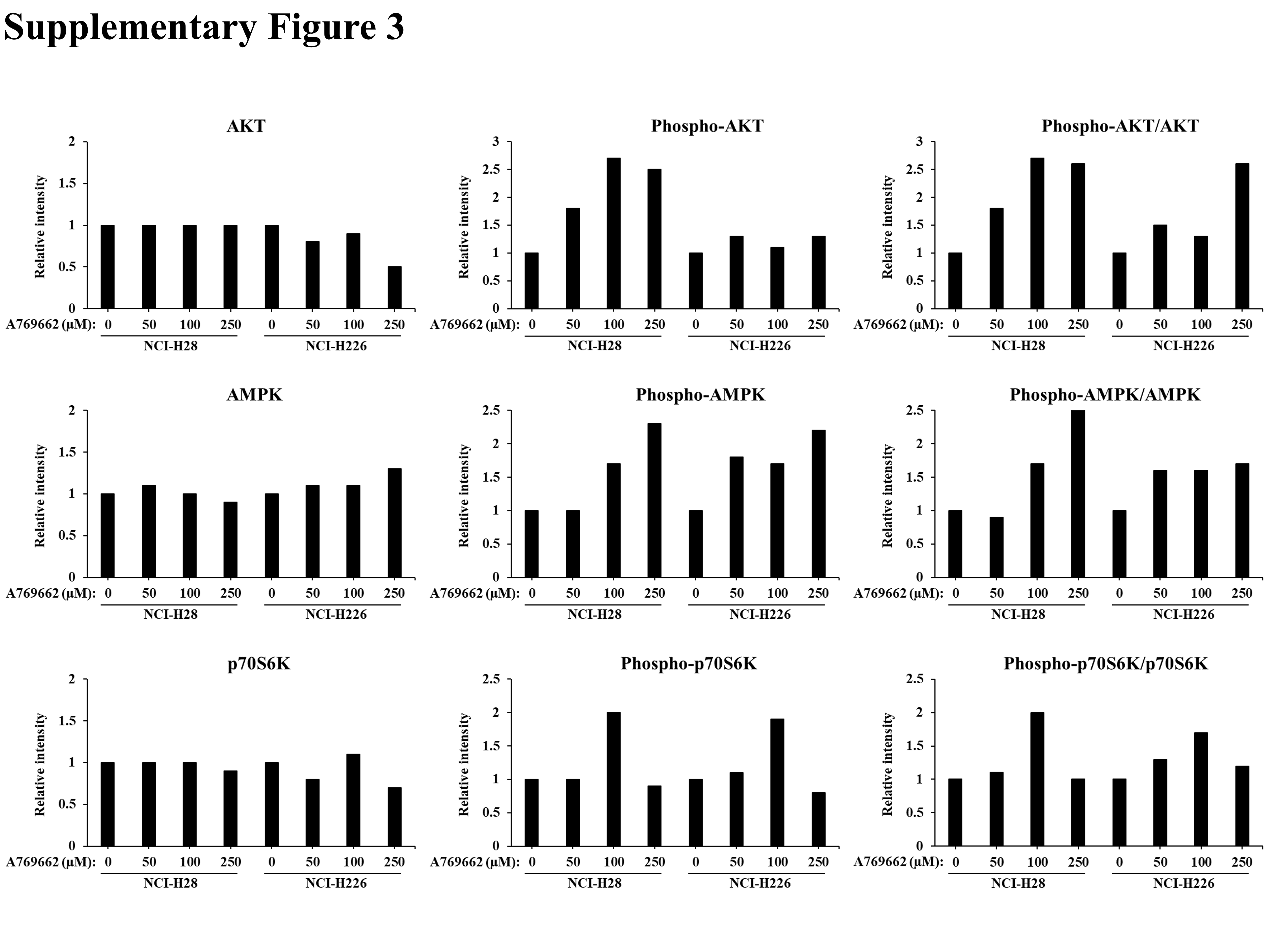

Supplement: Supplementary file 3 — Fig. S3. Relative expression levels of AKT, AMPK, p70S6K, and the respective phosphorylated proteins. Expression of these molecules in Figure 3B was quantitated as shown in Table S1 and expressed in bar graphs. A relative ratio between phosphorylated and total protein was also shown. [file MOL2-13-1419-s003.TIF]

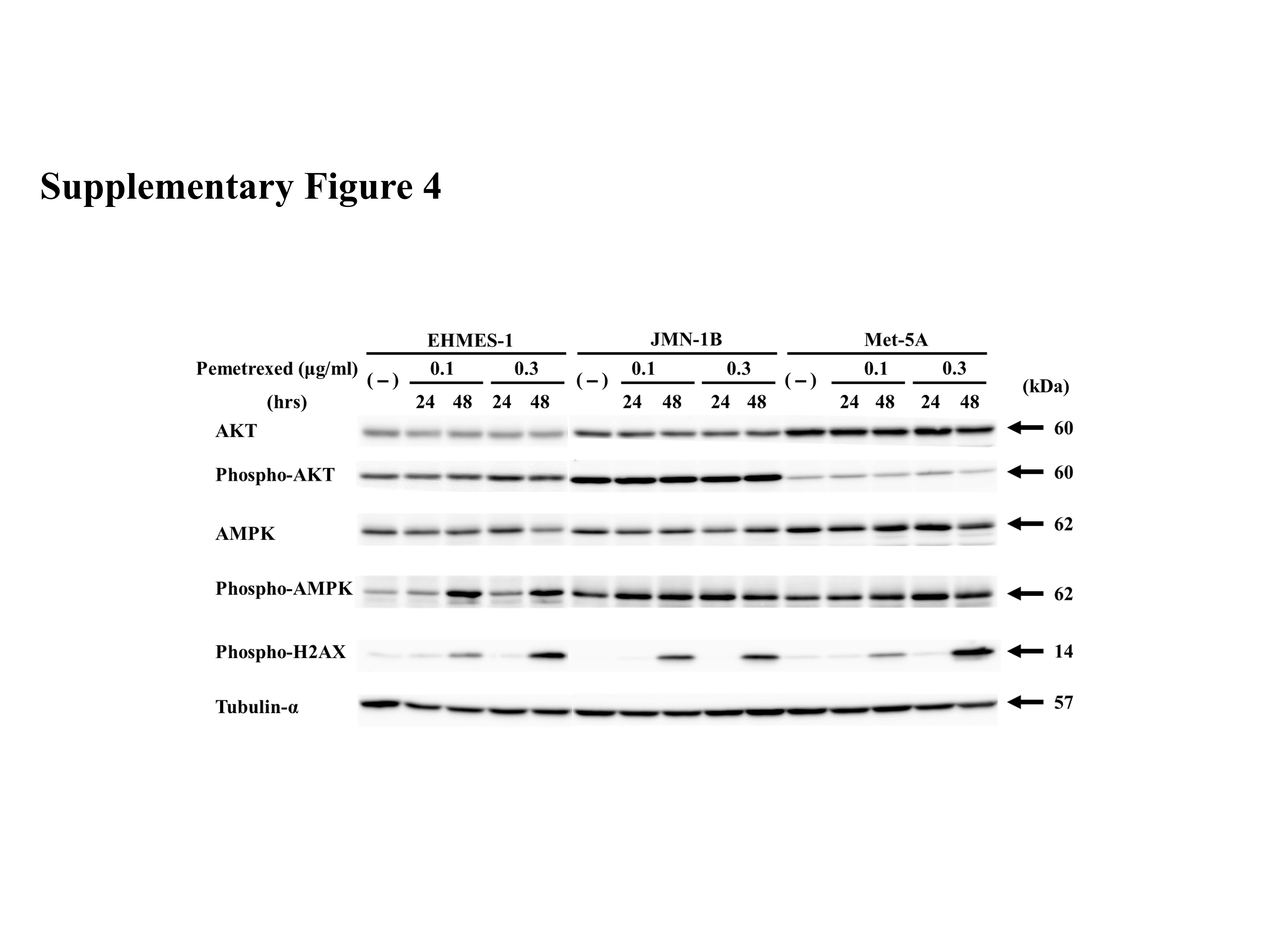

Supplement: Supplementary file 4 — Fig. S4. AMT and AMPK activation in PEM‐treated cells with mutated p53 genotype. Mesothelioma cells were treated with PEM as indicated and the cell lysate was subjected to Western blot analysis. Tubulin‐α was used as a loading control. [file MOL2-13-1419-s004.TIF]
