# Supplementary material for: AMPK activation induced in pemetrexed‐treated cells is associated with development of drug resistance independently of target enzyme expression
Source: Mol Oncol. 2019 May 15;13(6):1419–32. doi: 10.1002/1878-0261.12496 (PMC6547620; doi:10.1002/1878-0261.12496)
Supplement: Supplementary file 5 — Table S1. A relative expression level of major proteins in Western blots. Signal intensity of chemiluminescence was measured after subtraction of a background level with imagej software (National Institute of Health, Bethesda, MD, USA, available at https://imagej.nihgov/ij/index.html). Intensity is shown as an arbitrary unit standardized by control intensity (β‐actin or tubulin‐α). [file MOL2-13-1419-s005.docx]

**Supplementary Table 1**

| Figure 1B | NCI-H28 | | NCI-H226 | | MSTO-211H | | NCI-H2452 | |
| --- | --- | --- | --- | --- | --- | --- | --- | --- |
|  | parent | PEM | parent | PEM | parent | PEM | parent | PEM |
| AKT | 1 | 1.2 | 1 | 1.1 | 1 | 1.2 | 1 | 1.3 |
| Phospho-AKT | 1 | 2.5 | 1 | 0.7 | 1 | 0.8 | 1 | 10.3 |
| Phospho-AKT/AKT* | 1 | 2 | 1 | 0.6 | 1 | 0.7 | 1 | 8 |
| AMPK | 1 | 1 | 1 | 1.2 | 1 | 1 | 1 | 0.8 |
| Phospho-AMPK | 1 | 12 | 1 | 1.5 | 1 | 0.6 | 1 | 2.1 |
| Phospho-AMPK/AMPK* | 1 | 12.5 | 1 | 1.3 | 1 | 0.6 | 1 | 2.6 |
| 4E-BP1 | 1 | 0.9 | 1 | 1 | 1 | 1.2 | 1 | 1.2 |
| Phospho-4E-BP1 | 1 | 0.9 | 1 | 1 | 1 | 0.8 | 1 | 1.1 |
| Phospho-4E-BP1/4E-BP1* | 1 | 0.9 | 1 | 1 | 1 | 0.7 | 1 | 0.9 |
| p70S6K | 1 | 1 | 1 | 1.2 | 1 | 1.1 | 1 | 0.5 |
| Phospho-p70S6K | 1 | 1.5 | 1 | 1.5 | 1 | 0 | 1 | 1.2 |
| Phospho-p70S6K/p70S6K* | 1 | 1.5 | 1 | 1.3 | 1 | 0 | 1 | 2.4 |
| p53 | 1 | 2 | 1 | 1 | 1 | 1 | 1 | 1.2 |
| Phospho-p53 | 1 | 2.6 | 1 | 1 | 1 | 0.8 | 1 | 1 |
| Phospho-p53/p53* | 1 | 1.3 | 1 | 1.1 | 1 | 0.7 | 1 | 0.8 |

0: undetectable

| Figure 2A | NCI-H28 | | | | | H28-PEM | | | | |
| --- | --- | --- | --- | --- | --- | --- | --- | --- | --- | --- |
|  | (-) | PEM(0.1 µg/mL) | | PEM(0.3 µg/mL) | | (-) | PEM(0.1 µg/mL) | | PEM(0.3 µg/mL) | |
|  |  | 24 h | 48 h | 24 h | 48 h |  | 24 h | 48 h | 24 h | 48 h |
| AKT | 1 | 1.3 | 1.2 | 1.2 | 1.5 | 1 | 1.2 | 1.5 | 1.3 | 1.4 |
| Phospho-AKT | 1 | 1.7 | 1.8 | 1.6 | 2.4 | 1 | 1.2 | 1.8 | 1.5 | 1.3 |
| Phospho-AKT/AKT* | 1 | 1.3 | 1.4 | 1.3 | 1.6 | 1 | 1 | 1.2 | 1.2 | 1 |
| AMPK | 1 | 1.4 | 1.3 | 1.3 | 1.8 | 1 | 0.6 | 0.7 | 0.8 | 0.7 |
| Phospho-AMPK | 1 | 3.6 | 4.5 | 3.4 | 6.2 | 1 | 0.5 | 1 | 1 | 1 |
| Phospho-AMPK/AMPK* | 1 | 2.6 | 3.5 | 2.5 | 3.5 | 1 | 0.9 | 1.4 | 1.2 | 1.5 |
| 4E-BP1 | 1 | 1.4 | 1.2 | 1.2 | 1.3 | 1 | 1 | 1.1 | 1.1 | 1.2 |
| Phospho-4E-BP1 | 1 | 1.7 | 1.4 | 1.4 | 1.3 | 1 | 1.6 | 1.7 | 2 | 1.8 |
| Phospho-4E-BP1/4E-BP1* | 1 | 1.2 | 1.2 | 1.2 | 1 | 1 | 1.6 | 1.5 | 1.8 | 1.5 |
| p70S6K | 1 | 1.7 | 1.6 | 1.5 | 1.7 | 1 | 0.9 | 1.1 | 0.9 | 1 |
| Phospho-p70S6K | 1 | 3.2 | 2.5 | 3.1 | 3.5 | 1 | 2.2 | 4.2 | 2.7 | 3.2 |
| Phospho-p70S6K/p70S6K* | 1 | 1.9 | 1.6 | 2.1 | 2.1 | 1 | 2.3 | 3.8 | 3.1 | 3.2 |

| Figure 2B | NCI-H226 | | | | | H226-PEM | | | | |
| --- | --- | --- | --- | --- | --- | --- | --- | --- | --- | --- |
|  | (-) | PEM(0.1 µg/mL) | | PEM(0.3 µg/mL) | | (-) | PEM(0.1 µg/mL) | | PEM(0.3 µg/mL) | |
|  |  | 24 h | 48 h | 24 h | 48 h |  | 24 h | 48 h | 24 h | 48 h |
| AKT | 1 | 1.1 | 1.2 | 1.2 | 1.1 | 1 | 0.8 | 1 | 0.8 | 1 |
| Phospho-AKT | 1 | 2 | 1.9 | 2.1 | 1.9 | 1 | 0.7 | 0.7 | 0.7 | 0.6 |
| Phospho-AKT/AKT* | 1 | 1.7 | 1.6 | 1.7 | 1.7 | 1 | 0.9 | 0.7 | 0.8 | 0.6 |
| AMPK | 1 | 1 | 0.7 | 1 | 1.1 | 1 | 0.9 | 1.3 | 0.7 | 0.9 |
| Phospho-AMPK | 1 | 1.6 | 2.4 | 2.2 | 1.9 | 1 | 1.9 | 1.9 | 1.7 | 2.1 |
| Phospho-AMPK/AMPK* | 1 | 1.6 | 3.7 | 2.2 | 1.7 | 1 | 2.1 | 1.5 | 2.5 | 2.3 |
| 4E-BP1 | 1 | 1.1 | 1.1 | 1.2 | 1.2 | 1 | 0.9 | 1.3 | 1 | 1.4 |
| Phospho-4E-BP1 | 1 | 1.4 | 2 | 1.7 | 1.6 | 1 | 1.2 | 1.6 | 1.2 | 1.5 |
| Phospho-4E-BP1/4E-BP1* | 1 | 1.3 | 1.8 | 1.5 | 1.3 | 1 | 1.3 | 1.2 | 1.1 | 1.1 |
| p70S6K | 1 | 1.2 | 1.3 | 1.3 | 1.1 | 1 | 0.9 | 0.9 | 0.6 | 0.8 |
| Phospho-p70S6K | 1 | 3.4 | 7 | 9.2 | 6.3 | 1 | 2 | 2.1 | 1 | 1.5 |
| Phospho-p70S6K/p70S6K* | 1 | 2.8 | 5.5 | 7.3 | 5.7 | 1 | 2.3 | 2.4 | 1.8 | 1.9 |

| Figure 3B | NCI-H28 | | | | NCI-H226 | | | |
| --- | --- | --- | --- | --- | --- | --- | --- | --- |
|  | A769662(µM) | | | | A769662(µM) | | | |
|  | 0 | 50 | 100 | 250 | 0 | 50 | 100 | 250 |
| AKT | 1 | 1 | 1 | 1 | 1 | 0.8 | 0.9 | 0.5 |
| Phospho-AKT | 1 | 1.8 | 2.7 | 2.5 | 1 | 1.3 | 1.1 | 1.3 |
| Phospho-AKT/AKT* | 1 | 1.8 | 2.7 | 2.6 | 1 | 1.5 | 1.3 | 2.6 |
| AMPK | 1 | 1.1 | 1 | 0.9 | 1 | 1.1 | 1.1 | 1.3 |
| Phospho-AMPK | 1 | 1 | 1.7 | 2.3 | 1 | 1.8 | 1.7 | 2.2 |
| Phospho-AMPK/AMPK* | 1 | 0.9 | 1.7 | 2.7 | 1 | 1.6 | 1.6 | 1.7 |
| p70S6K | 1 | 1 | 1 | 0.9 | 1 | 0.8 | 1.1 | 0.7 |
| Phospho-p70S6K | 1 | 1 | 2 | 0.9 | 1 | 1.1 | 1.9 | 0.8 |
| Phospho-p70S6K/p70S6K* | 1 | 1.1 | 2 | 1 | 1 | 1.3 | 1.7 | 1.2 |

| Figure 4B | H28-PEM | | | | | H226-PEM | | | | |
| --- | --- | --- | --- | --- | --- | --- | --- | --- | --- | --- |
|  | (-) | 0.75 µM Compound C | | 1.5 µM Compound C | | (-) | 0.75 µM Compound C | | 1.5 µM Compound C | |
|  |  | 24h | 48h | 24h | 48h |  | 24h | 48h | 24h | 48h |
| AKT | 1 | 0.7 | 0.8 | 0.8 | 0.7 | 1 | 1 | 1 | 0.9 | 1 |
| Phospho-AKT | 1 | 0.8 | 0.8 | 0.7 | 0.8 | 1 | 0.8 | 0.9 | 0.9 | 0.7 |
| Phospho-AKT/AKT* | 1 | 1.2 | 1 | 0.9 | 1.1 | 1 | 0.8 | 0.9 | 1 | 0.7 |
| AMPK | 1 | 0.9 | 0.8 | 1 | 0.8 | 1 | 0.8 | 1 | 0.7 | 0.9 |
| Phospho-AMPK | 1 | 1.5 | 0.7 | 1.4 | 1.2 | 1 | 0.6 | 1 | 0.5 | 1.2 |
| Phospho-AMPK/AMPK* | 1 | 1.6 | 0.9 | 1.5 | 1.4 | 1 | 0.7 | 1 | 0.8 | 1.3 |
| p70S6K | 1 | 1.1 | 1.1 | 1.2 | 0.9 | 1 | 1 | 1 | 0.9 | 1 |
| Phospho-p70S6K | 1 | 2.9 | 2.6 | 3.3 | 2.6 | 1 | 3 | 1.8 | 3.3 | 1.8 |
| Phospho-p70S6K/p70S6K* | 1 | 2.7 | 2.4 | 2.8 | 2.9 | 1 | 3.1 | 1.7 | 3.8 | 1.8 |

| Figure 5B | H28-PEM | | | | | H226-PEM | | | | |
| --- | --- | --- | --- | --- | --- | --- | --- | --- | --- | --- |
|  | (-) | 1 µM Rapamycin | | 3 µM Rapamycin | | (-) | 1 µM Rapamycin | | 3 µM Rapamycin | |
|  |  | 24h | 48h | 24h | 48h |  | 24h | 48h | 24h | 48h |
| AKT | 1 | 1.4 | 1.6 | 1.6 | 1.7 | 1 | 1.1 | 0.7 | 0.8 | 0.7 |
| Phospho-AKT | 1 | 1.6 | 1.5 | 2.1 | 1.9 | 1 | 3 | 0.6 | 2.9 | 0.6 |
| Phospho-AKT/AKT* | 1 | 1.1 | 1 | 1.3 | 1.2 | 1 | 2.8 | 0.8 | 3.8 | 0.8 |
| AMPK | 1 | 0.7 | 0.6 | 0.7 | 0.7 | 1 | 0.8 | 0.7 | 0.7 | 0.9 |
| Phospho-AMPK | 1 | 0.7 | 0.7 | 0.8 | 1 | 1 | 1.4 | 1.3 | 1.4 | 1.3 |
| Phospho-AMPK/AMPK* | 1 | 1 | 1.1 | 1.2 | 1.4 | 1 | 1.9 | 1.8 | 2 | 1.5 |
| 4E-BP1 | 1 | 0.8 | 1.7 | 2 | 2.2 | 1 | 1.2 | 1.1 | 1.1 | 1 |
| Phospho-4E-BP1 | 1 | 0.7 | 1.2 | 1.7 | 2.4 | 1 | 0.9 | 0.9 | 0.9 | 0.7 |
| Phospho-4E-BP1/4E-BP1* | 1 | 0.8 | 0.8 | 0.8 | 1.1 | 1 | 0.8 | 0.8 | 0.8 | 0.7 |
| p70S6K | 1 | 0.8 | 1.1 | 1.1 | 0.8 | 1 | 0.8 | 0.7 | 0.6 | 0.6 |
| Phospho-p70S6K | 1 | 0.1 | 0.1 | 0.2 | 0.2 | 1 | 0.1 | 0.1 | 0.1 | 0.1 |
| Phospho-p70S6K/p70S6K* | 1 | 0.1 | 0.1 | 0.2 | 0.3 | 1 | 0.1 | 0.1 | 0.2 | 0.2 |

| Figure 5D | H28-PEM | | | | H226-PEM | | | |
| --- | --- | --- | --- | --- | --- | --- | --- | --- |
|  | PF4708671(µM) | | | | PF4708671(µM) | | | |
|  | 0 | 5 | 10 | 20 | 0 | 5 | 10 | 20 |
| AKT | 1 | 1.2 | 0.9 | 0.9 | 1 | 0.9 | 0.9 | 0.8 |
| Phospho-AKT | 1 | 1.7 | 1.7 | 1.8 | 1 | 1.2 | 1.3 | 1.3 |
| Phospho-AKT/AKT* | 1 | 1.4 | 2 | 2.1 | 1 | 1.3 | 1.5 | 1.6 |
| AMPK | 1 | 1 | 0.8 | 0.7 | 1 | 1 | 1.1 | 0.9 |
| Phospho-AMPK | 1 | 1.3 | 1 | 1.4 | 1 | 1.4 | 1.7 | 1.6 |
| Phospho-AMPK/AMPK* | 1 | 1.3 | 1.3 | 1.8 | 1 | 1.4 | 1.6 | 1.6 |
| 4E-BP1 | 1 | 1.1 | 0.9 | 0.8 | 1 | 0.9 | 0.9 | 0.7 |
| Phospho-4E-BP1 | 1 | 0.9 | 0.7 | 0.8 | 1 | 1 | 1 | 0.9 |
| Phospho-4E-BP1/4E-BP1* | 1 | 0.8 | 0.8 | 1 | 1 | 1.1 | 1.1 | 1.3 |
| p70S6K | 1 | 1.8 | 1.7 | 1.5 | 1 | 1.7 | 2 | 1.8 |
| Phospho-p70S6K | 1 | 0.8 | 0.8 | 0.6 | 1 | 0.8 | 0.4 | 0.4 |
| Phospho-p70S6K/p70S6K* | 1 | 0.5 | 0.5 | 0.4 | 1 | 0.4 | 0.2 | 0.2 |

| Figure 6B | H28-PEM | | | | | H226-PEM | | | | |
| --- | --- | --- | --- | --- | --- | --- | --- | --- | --- | --- |
|  | (-) | 4 µM MK-2206 | | 8 µM MK-2206 | | (-) | 4 µM MK-2206 | | 8 µM MK-2206 | |
|  |  | 24h | 48h | 24h | 48h |  | 24h | 48h | 24h | 48h |
| AKT | 1 | 1.3 | 1.1 | 1.1 | 1.9 | 1 | 0.7 | 0.8 | 0.8 | 0.7 |
| Phospho-AKT | 1 | 0.3 | 0.5 | 0.3 | 0.9 | 1 | 0.3 | 0.8 | 0.4 | 0.7 |
| Phospho-AKT/AKT* | 1 | 0.2 | 0.4 | 0.3 | 0.4 | 1 | 0.5 | 1 | 0.4 | 1 |
| AMPK | 1 | 1.1 | 1 | 1 | 0.6 | 1 | 0.7 | 1 | 0.9 | 1 |
| Phospho-AMPK | 1 | 1 | 1.4 | 0.7 | 1 | 1 | 0.7 | 1 | 0.9 | 1 |
| Phospho-AMPK/AMPK* | 1 | 0.9 | 1.3 | 0.6 | 1.6 | 1 | 1 | 1.1 | 1 | 1.1 |
| 4E-BP1 | 1 | 2.5 | 2 | 2.7 | 0.7 | 1 | 0.8 | 1.1 | 1 | 1.1 |
| Phospho-4E-BP1 | 1 | 1.1 | 0.9 | 0.8 | 0.2 | 1 | 0.7 | 1.1 | 1 | 0.9 |
| Phospho-4E-BP1/4E-BP1* | 1 | 0.4 | 0.4 | 0.3 | 0.3 | 1 | 0.9 | 1 | 1 | 0.9 |
| p70S6K | 1 | 1 | 0.9 | 0.9 | 0.3 | 1 | 0.8 | 0.9 | 0.9 | 0.9 |
| Phospho-p70S6K | 1 | 0.6 | 0.7 | 0.4 | 0.3 | 1 | 0.6 | 1 | 0.8 | 1 |
| Phospho-p70S6K/p70S6K* | 1 | 0.6 | 0.8 | 0.4 | 0.9 | 1 | 0.7 | 1.1 | 0.9 | 1.1 |

| Figure 7B | NCI-H28 | | | | | | H28-PEM | | | | | |
| --- | --- | --- | --- | --- | --- | --- | --- | --- | --- | --- | --- | --- |
|  | (-) | DMSO | Nutlin-3a | | | | (-) | DMSO | Nutlin-3a | | | |
|  |  |  | 24h | | 48 h | |  |  | 24 h | | 48 h | |
|  |  |  | 20 µM | 50 µM | 20 µM | 50 µM |  |  | 20 µM | 50 µM | 20 µM | 50 µM |
| AKT | 1 | 0.9 | 1.2 | 1.1 | 1.3 | 1 | 1 | 0.8 | 0.9 | 0.6 | 0.9 | 0.5 |
| Phospho-AKT | 1 | 0.6 | 1.4 | 1.6 | 0.8 | 0.5 | 1 | 0.8 | 1.2 | 2.3 | 1.8 | 1.9 |
| Phospho-AKT/AKT* | 1 | 0.7 | 1.2 | 1.4 | 0.6 | 0.5 | 1 | 1 | 1.3 | 3.7 | 2 | 3.5 |
| AMPK | 1 | 1 | 1.2 | 1.1 | 1.1 | 0.9 | 1 | 0.9 | 1.1 | 1 | 1.1 | 0.8 |
| Phospho-AMPK | 1 | 1.2 | 3.7 | 2.8 | 3.7 | 4.1 | 1 | 1.9 | 1.8 | 3.6 | 2.8 | 3.4 |
| Phospho-AMPK/AMPK* | 1 | 1.3 | 3.1 | 2.5 | 3.3 | 4.8 | 1 | 2 | 1.7 | 3.4 | 2.6 | 4.3 |
| 4E-BP1 | 1 | 0.8 | 1.1 | 1.4 | 1 | 1.1 | 1 | 0.8 | 1.1 | 1.4 | 1 | 1.1 |
| Phospho-4E-BP1 | 1 | 1.5 | 1.1 | 1.6 | 0.6 | 1.4 | 1 | 0.7 | 0.9 | 0.9 | 1 | 0.7 |
| Phospho-4E-BP1/4E-BP1* | 1 | 1.8 | 1 | 1.1 | 0.6 | 1.2 | 1 | 0.8 | 0.8 | 0.6 | 1 | 0.6 |
| p70S6K | 1 | 0.9 | 0.9 | 0.9 | 0.7 | 0.4 | 1 | 1.1 | 0.9 | 0.7 | 0.6 | 0.4 |
| Phospho-p70S6K | 1 | 1 | 1.3 | 0.7 | 0.3 | 0.2 | 1 | 1.1 | 1.2 | 1.3 | 0.8 | 0.4 |
| Phospho-p70S6K/p70S6K* | 1 | 1.1 | 1.5 | 0.9 | 0.5 | 0.5 | 1 | 1 | 1.4 | 1.9 | 1.4 | 1.1 |

| Figure 7C | NCI-H226 | | | | | | H226-PEM | | | | | |
| --- | --- | --- | --- | --- | --- | --- | --- | --- | --- | --- | --- | --- |
|  | (-) | DMSO | Nutlin-3a | | | | (-) | DMSO | Nutlin-3a | | | |
|  |  |  | 24h | | 48 h | |  |  | 24 h | | 48 h | |
|  |  |  | 20 µM | 50 µM | 20 µM | 50 µM |  |  | 20 µM | 50 µM | 20 µM | 50 µM |
| AKT | 1 | 0.9 | 1.3 | 1 | 0.9 | 0.4 | 1 | 0.8 | 0.9 | 0.6 | 0.9 | 0.5 |
| Phospho-AKT | 1 | 1.4 | 1.3 | 1 | 0.9 | 0.3 | 1 | 0.7 | 1 | 0.3 | 1.1 | 0.6 |
| Phospho-AKT/AKT* | 1 | 1.6 | 1.1 | 1 | 1 | 0.7 | 1 | 0.8 | 1.1 | 0.5 | 1.2 | 1 |
| AMPK | 1 | 1 | 0.8 | 0.6 | 0.6 | 0.3 | 1 | 0.9 | 1.1 | 0.9 | 1 | 1 |
| Phospho-AMPK | 1 | 1.3 | 0.4 | 1.1 | 2.1 | 3.4 | 1 | 1.2 | 1 | 1.1 | 1.7 | 3.3 |
| Phospho-AMPK/AMPK* | 1 | 1.4 | 0.5 | 1.9 | 3.4 | 10.7 | 1 | 1.4 | 0.8 | 1.3 | 1.6 | 3.1 |
| 4E-BP1 | 1 | 0.9 | 1.2 | 1.7 | 1 | 0.9 | 1 | 1.6 | 2.2 | 3.1 | 1.9 | 3.1 |
| Phospho-4E-BP1 | 1 | 0.8 | 0.9 | 0.8 | 0.5 | 0.4 | 1 | 1 | 0.9 | 0.6 | 0.5 | 0.6 |
| Phospho-4E-BP1/4E-BP1* | 1 | 0.8 | 0.7 | 0.5 | 0.5 | 0.4 | 1 | 0.6 | 0.4 | 0.2 | 0.3 | 0.2 |
| p70S6K | 1 | 1 | 0.8 | 0.5 | 0.4 | 0.2 | 1 | 1 | 0.7 | 0.6 | 0.6 | 0.3 |
| Phospho-p70S6K | 1 | 1.1 | 0.8 | 0.8 | 0.5 | 0.3 | 1 | 0.8 | 0.8 | 0.9 | 0.5 | 1.2 |
| Phospho-p70S6K/p70S6K* | 1 | 1.1 | 1 | 1.5 | 1.4 | 1.5 | 1 | 0.8 | 1.1 | 1.6 | 1 | 3.5 |

| Supplementary Figure 1 | NCI-H28 | | MSTO-211H | | NCI-H2452 | |
| --- | --- | --- | --- | --- | --- | --- |
|  | parent | CDDP | parent | CDDP | parent | CDDP |
| AKT | 1 | 1.3 | 1 | 1 | 1 | 0.9 |
| Phospho-AKT | 1 | 7.4 | 1 | 2.5 | 1 | 3 |
| Phospho-AKT/AKT* | 1 | 5.5 | 1 | 2.5 | 1 | 3.3 |
| AMPK | 1 | 1.4 | 1 | 0.8 | 1 | 0.9 |
| Phospho-AMPK | 1 | 1.3 | 1 | 0.9 | 1 | 0.4 |
| Phospho-AMPK/AMPK* | 1 | 1 | 1 | 1.2 | 1 | 0.4 |

| Supplementary Figure 4 | EHMES-1 | | | | | JMN-1B | | | | | MeT-5A | | | | |
| --- | --- | --- | --- | --- | --- | --- | --- | --- | --- | --- | --- | --- | --- | --- | --- |
|  | (-) | PEM(0.1 µg/ml) | | PEM(0.3 µg/ml) | | (-) | PEM(0.1 µg/ml) | | PEM(0.3 µg/ml) | | (-) | PEM(0.1 µg/ml) | | PEM(0.3 µg/ml) | |
|  |  | 24 h | 48 h | 24 h | 48 h |  | 24 h | 48 h | 24 h | 48 h |  | 24 h | 48 h | 24 h | 48 h |
| AKT | 1 | 0.8 | 0.8 | 0.8 | 0.8 | 1 | 1 | 0.8 | 0.9 | 0.9 | 1 | 0.9 | 0.9 | 1 | 0.8 |
| Phospho-AKT | 1 | 0.9 | 1 | 1.3 | 1.2 | 1 | 1 | 1 | 1 | 1 | 1 | 1.1 | 0.9 | 1.1 | 0.7 |
| Phospho-AKT/AKT* | 1 | 1.2 | 1.2 | 1.7 | 1.5 | 1 | 1.1 | 1.1 | 1 | 1 | 1 | 1.2 | 1 | 1.1 | 0.8 |
| AMPK | 1 | 0.9 | 0.9 | 1.1 | 0.6 | 1 | 0.8 | 0.9 | 0.7 | 1 | 1 | 1 | 1.1 | 1.2 | 0.8 |
| Phospho-AMPK | 1 | 1.4 | 5.8 | 1.9 | 5.2 | 1 | 1.5 | 1.4 | 1.5 | 1.2 | 1 | 1.1 | 1.4 | 1.8 | 1.4 |
| Phospho-AMPK/AMPK* | 1 | 1.6 | 6.2 | 1.7 | 8.7 | 1 | 1.8 | 1.6 | 2.2 | 1.2 | 1 | 1.2 | 1.3 | 1.5 | 1.7 |

Intensity of parent cells was adjusted as 1.

* Relative ratio of phosphorylated protein to the total protein.

Signal intensity of chemiluminescence was measured after subtraction of a background level with ImageJ software (National Institute of Health, Bethesda, MD, USA, available at https://imagej.nih gov/ij/index.html). Intensity is shown as an arbitrary unit standardized by control intensity (β-actin or tubulin-α).
